# Differentiation of human hyalocytes from induced pluripotent stem cells through ascorbic acid treatment

**DOI:** 10.1007/s13577-025-01182-2

**Published:** 2025-02-12

**Authors:** Elena Laura Mazzoldi, Gabriele Benini, Rosalba Monica Ferraro, Moira Micheletti, Giovanni Martellosio, Viola Balduchelli, Piergiuseppe Sacristani, Daniele Lussignoli, Francesco Semeraro, Sara Rezzola, Marco Presta, Loredana Bergandi, Alessandro Meduri, Silvia Clara Giliani

**Affiliations:** 1 Department of Molecular and Translational Medicine, University of Brescia, Viale Europa 11, 25123 Brescia, Italy; 2 «Angelo Nocivelli» Institute for Molecular Medicine, ASST Spedali Civili, Piazzale Spedali Civili 1, 25123 Brescia, Italy; 3 Hematology Unit, Clinical Chemistry Laboratory, Diagnostic Department, ASST Spedali Civili of Brescia, Piazzale Spedali Civili 1, 25123 Brescia, Italy; 4 Department of Ophthalmology, University of Brescia, ASST Spedali Civili, Piazzale Spedali Civili 1, 25123 Brescia, Italy; 5 Department of Oncology, University of Torino, Via Santena 5 Bis, 10126 Turin, Italy; 6 Ophthalmology Clinic, Department of Biomedical and Dental Sciences and Morphofunctional Imaging, University of Messina, 98125 Messina, Italy; 7 Section of Medical Genetics and Cytogenetics, ASST Spedali Civili of Brescia, Piazzale Spedali Civili 1, 25123 Brescia, Italy; 8 National Center for Gene Therapy and Drugs based on RNA Technology – CN3, Brescia, Italy

**Keywords:** Hyalocytes, Induced pluripotent stem cells, Macrophages, Vitreous body, Ascorbic acid

## Abstract

Hyalocytes are macrophage-like cells residing in the eye vitreous cortex. Even though hyalocytes have been firstly described in the mid-Nineteenth century, they have been poorly explored. Recent researches highlighted hyalocyte involvement in both physiological and pathological processes of the vitreoretinal interface. Nonetheless, the majority of works involving hyalocyte cultures were carried out in animals, while fewer studies were performed on humans because their isolation requires vitrectomy. The aim of this study was to differentiate human induced pluripotent stem cells (iPSCs) into hyalocytes as a non-invasive method to continuously obtain cells. iPSCs were first differentiated into hematopoietic stem/progenitor cells (HSPCs) and then into macrophages. Macrophages were either left untreated (NT) or treated with ascorbic acid (AA) alone or combined with bFGF and/or TGF-β1. Additionally, macrophages were cultured in the presence of a pool of vitreous bodies from vitrectomies. Cells were analyzed for morphology and then for gene and protein expression through qRT-PCR, immunofluorescence, Western Blot, and flow cytometry. Similar to cells treated with the vitreous body, macrophages treated with AA alone or in combination with bFGF exhibited a more elongated shape compared to NT or cells treated with TGF-β1. Additionally, these treatments resulted in gene expression downregulation for *S100A4*, *S100A10*, *S100B*, and *CX3CR1*, while upregulating *COL6A1*, *HLA-DRA*, and *CD74*. At the protein level, S100B, CD14, and CD49d were downregulated with all treatments, while collagen VI and HLA-DR were upregulated. This work demonstrates that hyalocytes can be differentiated by treatment of iPSC-derived macrophages with ascorbic acid for a period of 21 days.

**Supplementary Information:**

The online version contains supplementary material available at 10.1007/s13577-025-01182-2.

## Introduction

The vitreous body is the biggest compartment of the eye, situated posterior to the lens and anterior to the retina [1]. It is filled with a viscoelastic, transparent gel made up of water, proteins, glycosaminoglycans, and metabolites, in particular type II, V/XI, VI, and IX collagens and hydrated hyaluronic acid [2]. Ascorbic acid, 33-times more concentrated than in the blood, plays an important role in preventing cataract formation and vitreous age-related degeneration [3]. Three areas can be distinguished: basal, central, and the cortex [1, 3].

The vitreous body is almost completely acellular, with the exception of some rare cells in the vitreous cortex: hyalocytes, fibroblasts, and macrophages [2]. About 90% of this population is represented by hyalocytes, accounting for 1–2 × 10^5^ cells/eye [2, 4]. From a morphological point of view, they are similar to macrophages [4, 5]; moreover, their hematopoietic origin was demonstrated in mouse models [6, 7].

In literature, a few works tried to highlight markers than could distinguish between macrophages and hyalocytes, but there is very poor consensus [5, 6, 8–10]. Nonetheless, it has been demonstrated that hyalocytes synthetize and degrade extracellular matrix (ECM) molecules [11–14], display phagocytic properties [4], secrete cytokines and enzymes [15, 16], and play a role in maintaining the vitreous transparent, avascular, and immunologically privileged [17, 18]. In a pathological scenario, they are involved in the formation of epiretinal membrane [19, 20] and of macular holes [21], in the proliferative diabetic retinopathy [22], and in the collagen gel contraction [23, 24].

Even though hyalocytes have been known since the mid-Nineteenth century [25], these cells have been poorly investigated. Indeed, the majority of studies on hyalocytes were conducted through immunohistochemical approaches and mainly on animal eyes (chick [11], quail [8], mouse [6, 10], rabbit [26], guinea pig [27], horse [5], bovine [15, 23], pig [12, 14, 28, 29]). In few studies, vital hyalocytes have been isolated from slaughtered animals and cultured [11, 12, 14, 23, 29].

Also in human settings, some investigations have been conducted on epiretinal membrane and internal limiting membrane specimens through immunocytochemistry, immunofluorescence and immunoelectron microscopy [19, 21, 30]. In a pioneeristic work, Kashiwagi and colleagues managed to establish human cell lines from hyalocytes isolated from surgical specimens and transformed through HPV16 E6 and E7 genes [9]; in 2020, Nuzzi and colleagues obtained primary human hyalocyte cultures from vitrectomies, that could proliferate in vitro for 1–3 months [31]. Recently, Micera *et al.* cultured human hyalocytes up to 6 days, which retained their in situ phenotype [24].

In the last years, Boneva and colleagues conducted transcriptomic investigations on FACS-sorted human hyalocytes and found that genes related to humoral immune response, leukocyte migration, and antigen presentation were more expressed in hyalocytes than in other myeloid populations [32]. On the other hand, similarities were demonstrated between hyalocytes and paired retinal microglia transcriptome in human enucleated eyes [33].

Altogether, all the studies involving hyalocytes from living human subjects required invasive procedures including eye enucleation [9, 33] or vitrectomy [31, 32]. Instead, the induced pluripotent stem cell (iPSC) technology could represent an important option to have an unlimited source of patient-specific hyalocytes in a non-invasive way, as it is already for macrophages [34] and other blood cells [35], mammary gland [36], neural [37, 38], skin [39], muscle [40], and other tissues’ cells. To our knowledge, the present work is the first report about the establishment of a differentiation protocol to obtain human hyalocytes from iPSCs.

## Materials and methods

### Human vitreous body collection

Sixty-nine vitreous body samples were obtained from patients undergoing *pars plana* vitrectomy at the Clinics of Ophthalmology (ASST Spedali Civili di Brescia), from November 2020 to October 2024 (Table 1). Samples were stored at − 80 °C until utilization. Pools were prepared from 4 to 6 samples according to the date of surgery.

**Table 1:** Anagraphic and clinical characteristics of patients undergoing *pars plana* vitrectomy

Gender (M/F)	37 (54%)/32 (46%)
Age	69.13±10.18
Macular pucker	41 (59.4%)
Macular hole	15 (22%)
Vitreomacular traction	4 (6%)
Dislocated intraocular lens	1 (1.4%)
Foveoschisis	1 (1.4%)
Crystalline deposits in vitreous	1 (1.4%)
Retinal detachment	5 (7%)
Lamellar hole	1 (1.4%)

### *In situ* vitreous cell isolation

Cells have been isolated from three patients undergoing scleral fixation intraocular lens (IOL) surgery at the Gaetano Martino University Hospital of Ophthalmology, Messina (Italy).

During scleral fixation IOL, an anterior vitrectomy was performed, given the presence of vitreous content in the anterior chamber due to the absence of the posterior capsule. Generally, 1 mL sample of vitreous was collected from each patient. Samples were immediately centrifuged at 1,380 × g for 10 min. The clear supernatant was discarded, whereas the cellular component and debris (in situ vitreous cells) were washed with PBS, and total RNA was extracted with the Cells-to-CT™ 1-Step PowerSYBR^®^ Green Kit (Ambion; Thermo Fisher Scientific, Waltham, MA) as previously described [41, 42] and following the manufacturer’s instruction.

### Induced pluripotent stem cell culture

Two iPSC lines have been used: BJ (clone 2, 21, and 23) and Episomal. The BJ cell line was obtained by reprogramming human healthy neonatal fibroblasts (ATCC, Manassas, VA) by using the CytoTune™-iPS 2.0 Sendai Reprogramming Kit (Invitrogen ™, Thermo Fisher Scientific). Human Episomal cell line is a commercial iPSC line obtained from cord blood-derived CD34^+^ progenitors (Gibco^™^, Thermo Fisher Scientific).

iPSCs were cultured on hESC-qualified Matrigel^®^ (Corning, Corning, NY) in NutriStem^®^ hPSC XF medium (Biological Industries, Kibbutz Beit Haemek, Israel) supplemented with 10 ng/mL basic fibroblast growth factor (bFGF, Merck Millipore, Burlington, MA), at 37 °C in a humidified incubator with 5% CO_2_, as previously described [43]. Medium was changed every day and colonies were passaged by manual picking every 5 days.

### Differentiation of iPSCs in hematopoietic stem/progenitor cells (HSPCs)

STEMdiff^™^ Hematopoietic Kit (STEMCELL Technologies, Vancouver, Canada) was used as per manufacturer instructions for HSPC differentiation. Briefly, cells have been cultured on Matrigel^®^ for 3 days in medium A and 9 days in medium B, with half-medium change at day 2, 5, 7, and 10. HSPCs were collected from the supernatant for subsequent analyses and differentiation. Pictures were taken with an inverted microscope (EVOS™ XL, Thermo Fisher Scientific).

### Peripheral blood mononuclear cell (PBMC) isolation

Peripheral blood from healthy volunteers was collected in lithium-heparin tubes and PBMCs were separated by density gradient centrifugation by using Lympholyte^®^ (Cedarlane Labs, Burlington, ON).

### Differentiation of macrophages from HSPCs and PBMCs

HSPCs and monocytes from PBMCs were differentiated into macrophages by culturing in macrophage medium, *i.e.* Iscove’s Modified Dulbecco’s Medium (IMDM, Euroclone, Milan, Italy) supplemented with 10% fetal bovine serum (FBS, Euroclone), 1% penicillin–streptomycin (Euroclone), 1% L-glutamine (Euroclone), 100 μM β-mercaptoethanol (Gibco^™^, Thermo Fisher Scientific), and 100 ng/mL Macrophage Colony-Stimulating Factor (M-CSF, Peprotech, Cranbury, NJ) at 37 °C, 5% CO_2_, in a humidified incubator. Cell culture has been carried out for 21 days: half medium change was performed twice a week and cells were analyzed at day 14 and 21.

### Film fixation, May–Grünwald-Giemsa staining, and examination

Following air-drying, thin films were fixed in absolute ethanol for 2 min. The same procedure was applied for cells carried on slides and then covered by a coverslip.

Films were stained by immersion in undiluted May–Grünwald stain for 2 min, followed by Giemsa’s stain for 4 min. Films were then washed in water and let dry in air.

Films were examined with 60 × and 100 × oil immersion objectives using a BX53 Olympus optical microscope and photographed with an Olympus SC180 camera. The measurements have been performed with Olympus software Cell Sens.

### Differentiation of macrophages in hyalocytes

Day 21-macrophages obtained from iPSCs have been cultured in complete macrophage medium supplemented with 100 μg/mL L-ascorbic acid (AA, Merck Millipore), with or without the addition of 10 ng/mL bFGF (Merck Millipore) and/or 10 ng/mL transforming growth factor β1 (TGF-β1, Peprotech). As negative control, macrophages were left untreated (NT) while, as positive control of differentiation, cells were cultured in presence of a pool of vitreous fluids from vitrectomy, at 1:4 dilution [44]. Half-medium change was performed every 2 days and cells were analyzed at day 7, 14, and 21.

### RNA extraction, reverse transcription, and quantitative real time PCR

Total RNA was extracted using NucleoSpin^®^ kit (Macherey–Nagel, Dueren, Germany) and quantified at Infinite^®^ M200 spectrophotometer (TECAN, Männedorf, Switzerland). DNase treatment was performed with TURBO DNA-free™ kit (Ambion, Thermo Fisher Scientific) to remove genomic DNA. RNA was then retrotranscribed into cDNA using ImProm-II^™^ Reverse Transcription System kit (Promega, Madison, WI). Gene expression was evaluated in qRT-PCR by mixing 10 ng cDNA with the iTaq Universal SYBR Green Supermix (Bio-Rad, Hercules, CA) and the gene-specific primers; each sample was run in duplicate. Primers are listed in Table 2 (IDT, Coralville, IA).

**Table 2:** qRT-PCR primer list

Gene	Primer forward (5′→ 3′)	Primer reverse (5′→ 3′)
*CD163*	TTTGTCAACTTGAGTCCCTTCAC	TCCCGCTACACTTGTTTTCAC
*FCGR1*	ATACAGGTGCCAGAGAGGTCTC	CCAGCTTATCCTTCCACGCATG
*CD68*	TGGGGCAGAGCTTCAGTTG	TGGGGCAGGAGAAACTTTGC
*ITGAM*	GCCTTGACCTTATGTCATGGG	CCTGTGCTGTAGTCGCACT
*VIM*	GGACCAGCTAACCAACGACA	AAGGTCAAGACGTGCCAGAG
*PLAU*	TCGTGAGCGACTCCAAAGGCA	GGGCAGTTGCACCAGTGAATGT
*CX3CR1*	ACTTTGAGTACGATGATTTGGCT	GGTAAATGTCGGTGACACTCTT
*FTL*	CAGCCTGGTCAATTTGTACCT	GCCAATTCGCGGAAGAAGTG
*SPP1*	GAAGTTTCGCAGACCTGACAT	GTATGCACCATTCAACTCCTCG
*CD74*	CCGGCTGGACAAACTGACA	GGTGCATCACATGGTCCTCTG
*CD46*	CGAGTGTCCCTTTCCTTCCT	CATAGCTTCAAATGTTGGTGGC
*CD86*	GAAACTGACAAGACGCGGC	CCAAGGAATGTGGTCTGGG
*HLA-DRA*	AGTCCCTGTGCTAGGATTTTTCA	ACATAAACTCGCCTGATTGGTC
*S100A4*	CCCTGGATGTGATGGTGTCC	TTGTCCCTGTTGCTGTCCAA
*S100A10*	ACATTTCACAAATTCGCTGGG	AGCCCACTTTGCCATCTCTAC
*S100B*	GAAGAAATCCGAACTGAAGGAGC	TCCTGGAAGTCACATTCGCCGT
*COL1A2*	AGTGGTTACTACTGGATTGACC	TTGCCAGTCTCCTCATCC
*COL2A1*	TGATGAAAAGGCTGGTGGCG	CTGACCATCGTTGCCTCGGG
*COL6A1*	ACACCGACTGCGCTATCAAG	CGGTCACCACAATCAGGTACTT
*COL6A3*	GAAGACCGGCAGCTCATCAA	CGATGTTGCAGATGTCCAAGCA
*COL9A1*	GGCAGTAGAGGAGAATTAGGACC	GTTCACCGACTACACCCCTG
*COL11A1*	GTCCTCCAGGTCTACAAGGC	ACGGAACGGTAACATCAACATAG
*HAS1*	CTGCGATACTGGGTAGCCTTCA	CCAGGAACTTCTGGTTGTACCAG
*HAS2*	GTCATGTACACAGCCTTCAGAGC	ACAGATGAGGCTGGGTCAAGCA
*HAS3*	AGCACCTTCTCGTGCATCATGC	TCCTCCAGGACTCGAAGCATCT
*POMC*	CTCACCACGGAAAGCAACC	AAGTGGCCCATGACGTACTT
*B2M*	TCTCTCTTTCTGGCCTGGAG	TCTCTGCTGGATGACGTGAG

qRT-PCR was performed on CFX96 C1000 Touch^™^ Real-Time PCR Detection System (Bio-Rad). PCR amplification was conducted with 1 cycle of denaturation at 98 °C for 30 s and 45 cycles of amplification, including denaturation at 95 °C for 5 s and annealing/extension at 60 °C for 30 s. The specificity of the PCRs was confirmed with melt curve analysis. Nonspecific amplifications were never detected. The relative quantification was obtained with the 2^−ΔΔCt^ method, using either β-actin (*ACTB*, PrimeTime^®^ qPCR Primer Hs.PT.56a.19461448.g, IDT) or β2-microglobulin (*B2M*) as housekeeping gene and either HSPCs or NT samples as reference.

### Flow cytometry

Cells were stained with 100 ng/mL DAPI (Sigma Aldrich, St Louis, MO) to discriminate living cells, treated with FcR blocking reagent (Miltenyi Biotec, Bergisch Gladbach, Germany), and then incubated with the specific conjugated antibodies listed in Table 3. Not-labelled cells were used to set the autofluorescence.

**Table 3:** List of antibodies used in flow cytometry

Anti-human antibody	Fluorochrome	Brand	Dilution
CD45	APC	Miltenyi Biotec	1:50
CD34	PE	BD	1:25
CD43	FITC	BD	1:50
HLA-DR	PerCP-Cy5.5	BD	1:10
CD14	FITC	BioLegend	1:25
CD11b	APC	Miltenyi Biotec	1:100
CD16	PE	BD	1:10
CD49d	PE	eBioscience	1:20

The acquisition was performed on FACSCanto™ II (BD, Franklin Lakes, NJ) from at least 10^4^ events/tube. The data were elaborated with FlowJo software (TreeStar, Ashland, OR). The results are expressed either as percentage or as the difference between the mean fluorescence intensity of labelled cells and of the corresponding not-labelled control (ΔMFI), normalized on the ΔMFI of NT samples, according to the following formula:

$$\Delta {\text{MFI ratio}} = \frac{{\left( {\text {MFI} _{{{\text{labelled}}}} - \text {MFI} _{{{\text{unlabelled}}}} } \right)_{{{\text{treat}}}} }}{{\left( {\text {MFI} _{{{\text{labelled}}}} - \text {MFI} _{{{\text{unlabelled}}}} } \right)_{{{\text{NT}}}} }}$$

### Western blot (WB)

Cells were lysed with RIPA buffer (Merck Millipore) supplemented with Halt^™^ protease and phosphatase inhibitor cocktail and EDTA (Thermo Fisher Scientific). Protein concentration was determined by Bradford assay (Bio-Rad). Ten micrograms of protein were run on NuPAGE^™^ 4–12% Bis–Tris polyacrylamide gels (Invitrogen, Thermo Fisher Scientific) in denaturing and reducing conditions. Proteins were then transferred onto PVDF membranes (GE Healthcare, Chicago, IL). Membranes were blocked with 5% non-fat milk in TBST and incubated with primary antibodies overnight at 4 °C. The following primary antibodies, diluted in 5% BSA-TBST, were used: mouse anti-human S100B 1:500 (Santa Cruz Biotechnology) and mouse anti-human α-tubulin 1:5000 (Sigma Aldrich). After washing, membranes were incubated with secondary horseradish peroxidase (HRP)-conjugated anti-mouse IgG antibody (GE Healthcare), diluted 1:7000 in 1% milk-TBST, for 1 h at room temperature. After washing, the signal was detected with Westar ηC Ultra chemiluminescent substrate (Cyanagen, Bologna, Italy) on iBright CL1000 Imaging System (Invitrogen, Thermo Fisher Scientific), and band densitometry was analyzed on iBright analysis software (Thermo Fisher Scientific). S100B signal intensity was normalized to α-tubulin; NT sample was used as reference.

### Immunofluorescence

Cells seeded on coverslips were fixed and permeabilized with FIX & PERM^®^ kit (Nordic MUbio, Susteren, The Netherlands), and blocked with 5% BSA in PBS.

Cells have been incubated with primary antibody, rabbit anti-human COL6A1 1:100 (Abcam, Cambridge, UK), for 2 h at room temperature, then with secondary antibody Alexa Fluor^™^ 488 goat anti-rabbit IgG (H + L) 1:500 (Invitrogen, Thermo Fisher Scientific) for 1 h in the dark and finally with 100 ng/mL DAPI for 5 min.

Coverslips were mounted with ProLong^™^ Gold Antifade mountant (Invitrogen, Thermo Fisher Scientific), and were observed with an Olympus IX70 inverted fluorescence microscope (Olympus, Tokyo, Japan). Images were acquired by using Image-Pro Plus v7.0 software (Media Cybernetics, Rockville, MD).

### Statistical analysis

Experiments were repeated at least three times. Data were expressed as mean ± standard deviation. Comparisons between groups were performed using Student’s *t*-test on GraphPad Prism software (San Diego, CA).

## Results

### Induced pluripotent stem cells (iPSCs) were differentiated into hematopoietic stem/progenitor cells (HSPCs) by using a commercial kit

**Fig. 1:**
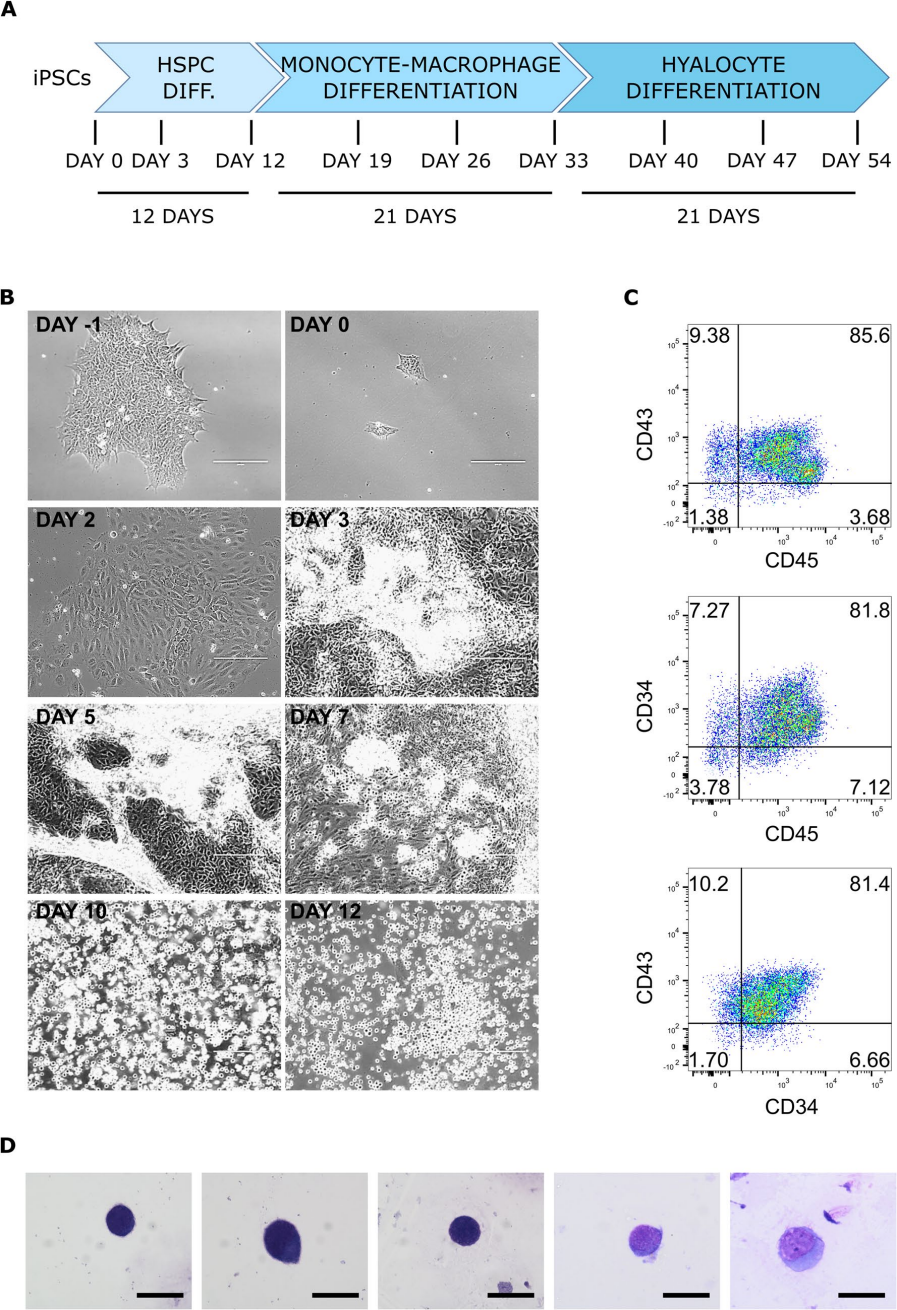
HSPC differentiation from iPSCs.** a** Scheme of the hyalocyte differentiation protocol, consisting in 12 days of HSPC differentiation, 21 days of monocyte-macrophage differentiation, and 21 days of hyalocyte differentiation. **b** Representative pictures of iPSC-to-HSPC differentiation steps. Scale bar: 200 µm. **c** Flow cytometry analysis of HSPC marker expression after 12 days of differentiation. Representative dot plots are shown. **d** May-Grünwald-Giemsa staining of smears obtained from HSPCs differentiated from iPSCs. Scale bar: 20 μm

We set up a 54 day-protocol to differentiate iPSCs into hyalocytes, which consists in three phases: HSPC differentiation (12 days), macrophage differentiation from HSPCs (21 days), followed by macrophage culture under different investigated stimuli (21 days; Fig. 1a).

For HSPC differentiation, we took advantage of StemDiff^™^ Hematopoietic kit (STEMCELL Technologies), which allows a robust and reproducible differentiation from iPSCs, without requiring either embryoid body (EB) generation or cocultures. A simple, 12-day protocol was followed to obtain suspension HSPCs from a supportive monolayer [35]. The main morphological changes observed are shown in Fig. 1b. At day 12, HSPCs could be collected from the supernatant.

To assess the quality of the obtained HSPCs, both flow cytometry and a cytological analysis have been carried out.

The flow cytometry analysis of the pan-leukocyte marker CD45 and of the HSPC markers CD34 and CD43 in suspension cells revealed a percentage of expression > 80% in all the experiments carried out (Fig. 1c); among adherent cells, cells positive for the three markers could also be found, but the percentage of expression was far lower (30–40%, data not shown).

The cytological staining revealed small, immature, mononucleated, highly basophil elements which resembled blast cells (Fig. 1d).

### HSPCs were differentiated into macrophages similar to blood-derived macrophages

BJ and Episomal HSPCs have been cultured in the presence of M-CSF for monocyte–macrophage differentiation and were characterized after 14 and 21 days of culture. As a comparison, PBMCs from healthy volunteers have been culture in the same conditions: monocytes got attached to the plastic surface and differentiated into macrophages.

**Fig. 2:**
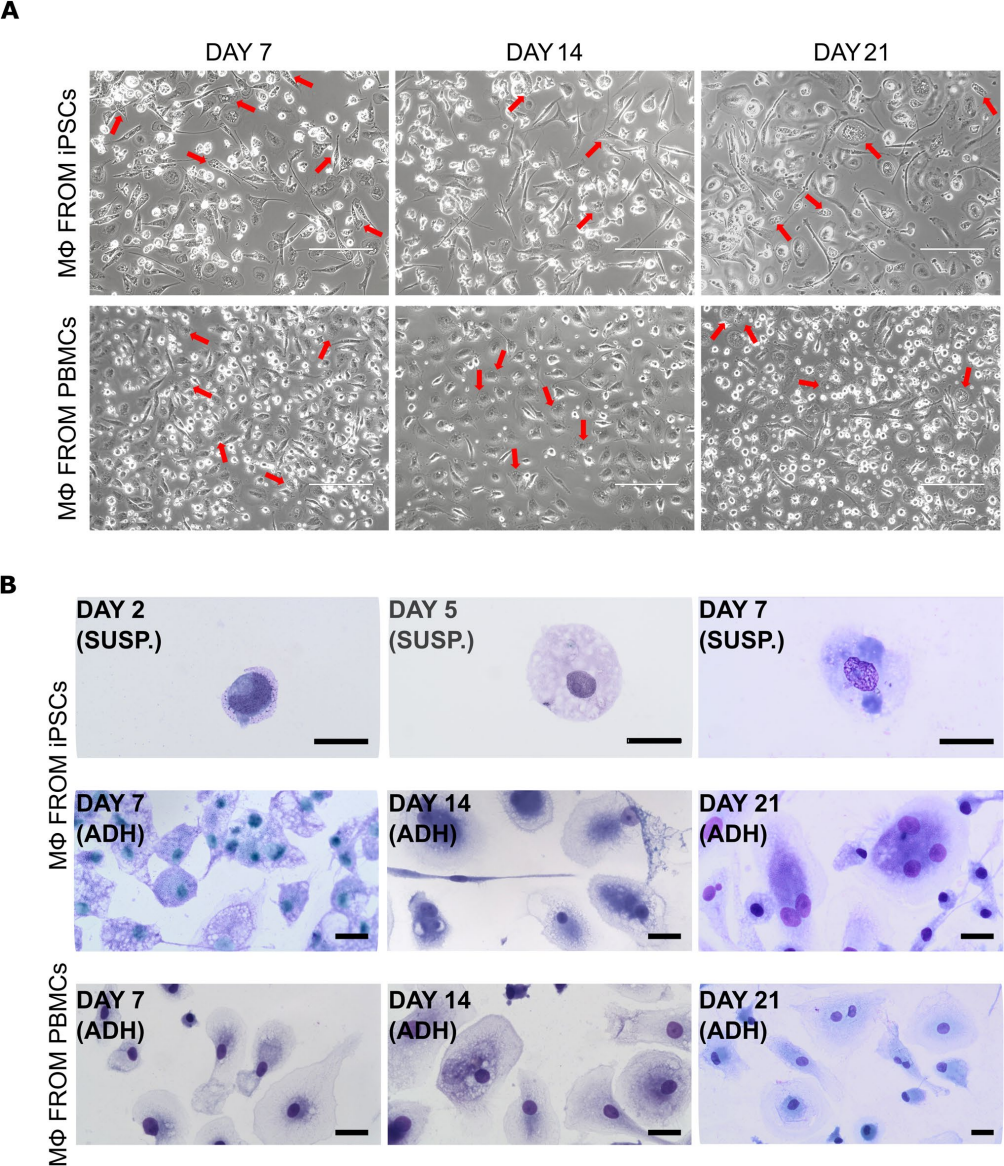
Macrophage differentiation from HSPCs. **a** Representative pictures of macrophages differentiated from iPSC-derived HSPCs and from PBMCs isolated from healthy volunteers’ blood, at day 7, 14, and 21. Scale bar: 200 µm. Red arrows indicate intracytoplasmic granules. **b** May-Grünwald-Giemsa staining of smears obtained from HSPCs at the first steps of differentiation towards monocytes (day 2, 5, and 7), and of adherent macrophages from iPSCs and from healthy donors’ PBMCs at day 7, 14, and 21. Scale bar: 20 μm

From a morphological point of view, cells from iPSCs and PBMCs presented similar features: attached cells were heterogeneous in shape, some were short and oval, some were elongated and rich in pseudopodia or lamellipodia. They tended to aggregate in small groups. In some of them, intracytoplasmic granules could be observed (Fig. 2a).

The cytological staining of cells at the first steps of myeloid differentiation (day 2, 5, and 7) revealed mononucleated elements of increased size with vacuolated and foamy cytoplasm, similar to the monocytes in cavitary liquids. At longer timepoints (day 7, 14, and 21), adherent cells appeared increased in size, sometimes of spindle-like shape, with vacuolated cytoplasm and very regular nuclei, compatible with the monocyte-macrophage lineage; sometimes cells were polynucleated. The cells differentiated from PBMCs also presented a macrophagic aspect, with abundant cytoplasm and sometimes more than one nucleus (Fig. 2b).

**Fig. 3:**
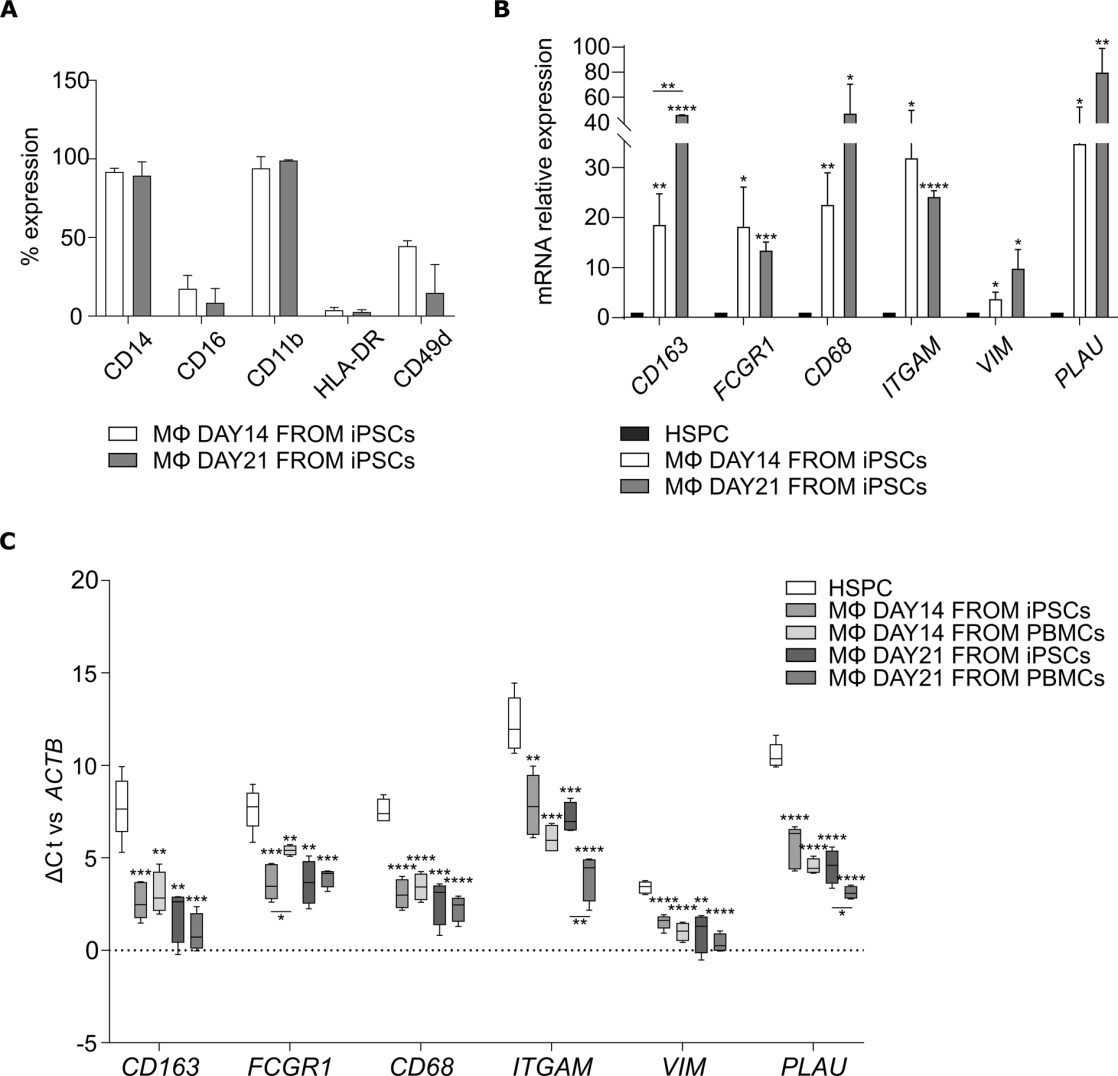
Macrophage characterization. **a** Flow cytometry analysis of macrophage marker expression at day 14 and 21 of differentiation from HSPCs. The bars represent the mean ± standard deviation of the percentage of positive cells (*n* = 3: BJ *n* = 2 and Episomal *n* = 1). **b** qRT-PCR analysis of macrophage-associated genes at day 14 and 21 of differentiation from HSPCs. Data were normalized to the corresponding HSPC of origin. The bars represent the mean ± standard deviation (*n* = 3: BJ *n* = 2 and Episomal *n* = 1). *p < 0.05, **p < 0.01, ***p < 0.001, ****p < 0.0001. **c** Box plots comparing the Δ cycle threshold of macrophage-associated genes versus *ACTNB* in HSPCs (*n* = 5), macrophages from iPSCs day 14 (*n* = 5: BJ *n* = 3 and Episomal *n* = 2) and day 21 (*n* = 4: BJ *n* = 3 and Episomal *n* = 1), and macrophages from healthy donors’ PBMCs (day 14 and 21, *n* = 4). *p < 0.05, **p < 0.01, ***p < 0.001, ****p < 0.0001

Flow cytometry analysis of macrophage surface markers at day 14 and 21 showed that almost 100% of iPSC-derived macrophages expressed CD14 and CD11b, while CD16, HLA-DR, and CD49d expression was variable (Fig. 3a).

Gene expression analysis of macrophage-associated genes showed a significant increase of *CD163*, *FCGR1*, *CD68*, *ITGAM*, *VIM*, and *PLAU* in iPSC-derived macrophages in comparison to HSPCs. The increase was more pronounced at day 21, except for *FCGR1* and *ITGAM* (Fig. 3b).

In order to demonstrate that iPSC-derived macrophages are similar to PBMC-derived macrophages at the transcriptional level, a comparative analysis of gene expression patterns was conducted among HSPCs and the aforementioned two groups at two distinct timepoints. To this end, the Δ cycle thresholds (Ct) versus *ACTNB* gene were calculated for all the considered markers (*CD163*, *FCGR1*, *CD68*, *ITGAM*, *VIM*, and *PLAU*), and compared among the groups. The ΔCt comparison was selected *in* *lieu* of the 2^−ΔΔCt^ method since, differently from iPSC-derived macrophages for which a specific parental HSPC can be identified (see Fig. 3b), a reference HSPC would be arbitrarily chosen for blood-derived macrophages, which would consequently influence the experimental outcome. Furthermore, the ΔCt comparison allows for the appreciation of the variability detected among the experimental replicates, and for the direct observation of the actual gene expression levels, rather than just a relative expression. Figure 3c highlights that all the macrophages (iPSC-derived and PBMC-derived) formed a cluster according to the ΔCt of each target, and were significantly different from HSPCs, which exhibited a higher ΔCt (*i.e.*, lower expression) for all genes.

### Characterization of mRNA expression in human *in situ* vitreous cells and in vitreous-treated macrophages from iPSCs

**Fig. 4:**
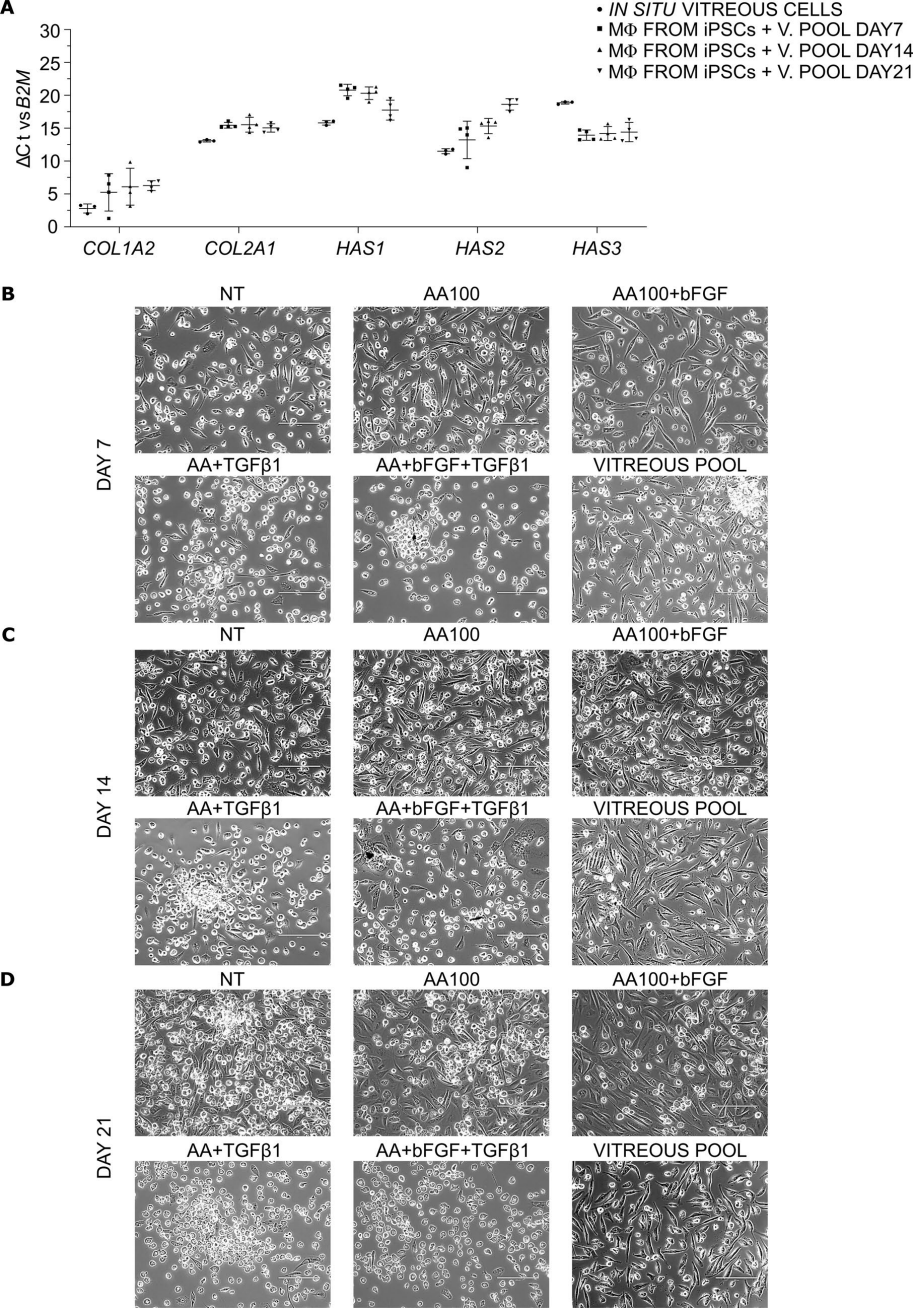
Hyalocyte differentiation from day 21-macrophages. **a** Scatter plots comparing the Δ cycle threshold of *COL1A2*, *COL2A1*, *HAS1*, *HAS2*, and *HAS3* genes versus *B2M* in in situ vitreous cells (*n* = 3) and in macrophages from iPSCs cultured in the presence of a pool of vitreous bodies (1:4) for 7, 14, and 21 days (*n* = 4: BJ *n* = 3 and Episomal *n* = 1). Individual values are reported. The horizontal bars represent the mean ± standard deviation. **b, c, d** Representative pictures of macrophages either not treated (NT) or treated with 100 µg/mL ascorbic acid (AA100) alone or in combination with 10 ng/mL bFGF and/or 10 ng/mL TGF-β1. Macrophages have also been cultured in the presence of a pool of vitreous bodies (1:4). Pictures were taken at day 7 (**b**), day 14 (**c**), and day 21 (**d**). Scale bar: 200 µm

Given that hyalocytes are supposed to produce the collagen and hyaluronic acid which constitute the vitreous body gel network, a comparative analysis of the gene expression of *COL1A2*, *COL2A1*, and of the three hyaluronan synthase isoforms *HAS1*, *HAS2*, and *HAS3*, was conducted in in situ vitreous cells from three patients and in day 21 iPSC-derived macrophages cultured in the presence of a pool of vitreous body for 7, 14, and 21 days, which served as the internal positive control of the differentiation protocol objective of this study. In all the samples, mRNAs were detected for the specified targets. Intriguingly, high expression levels of *COL1A2* were observed both in in situ vitreous cells and in macrophages from iPSCs treated with the vitreous pool (Ct < 26); in contrast, much lower expression levels were recorded for *COL2A1*, *HAS1*, *HAS2*, and *HAS3* (Ct > 30, Fig. 4a). Similar trends were found in both vitreous cells from patients, which are *bona fide* hyalocytes, and in iPSC-derived macrophages cultured with vitreous pools, meaning that the latter could represent a reliable model for hyalocyte differentiation.

### iPSC-derived macrophages have been treated with ascorbic acid, bFGF, and/or TGF-β1 to obtain hyalocytes

Based on the existing literature on porcine and human hyalocyte culture, we cultured iPSC-derived macrophages (day 21) in the presence of 100 μg/mL AA alone or in combination with 10 ng/mL bFGF and/or 10 ng/mL TGF-β1.

Cells have been analyzed at day 7, 14, and 21 for morphology, gene, and protein expression.

Pictures in bright field showed a more elongated morphology in vitreous-treated macrophages, as well as in AA- and AA + bFGF-treated cells, than in NT, where heterogeneous morphologies were observed; on the contrary, TGF-β1-treated cells were round-shaped (Fig. 4b, c, d). The differences in cell morphology were more pronounced at day 14 (Fig. 4c) and at day 21 (Fig. 4d).

Phalloidin fluorescence staining was performed to highlight any differences in thickness or distribution of actin filaments, as previously demonstrated elsewhere [14, 31]. Unfortunately, we could not observe any significant difference, but the staining was still useful to confirm the different morphology (Suppl. Figure 1, 2, and 3).

### iPSC-derived hyalocytes showed a different expression of several genes in comparison to NT macrophages

To further characterize iPSC-derived macrophages after treatments, qRT-PCR analysis was performed on a group of genes that have been described as up or downregulated in hyalocytes as compared to macrophages [5, 8, 9, 12, 15, 23, 30, 32].

**Fig. 5:**
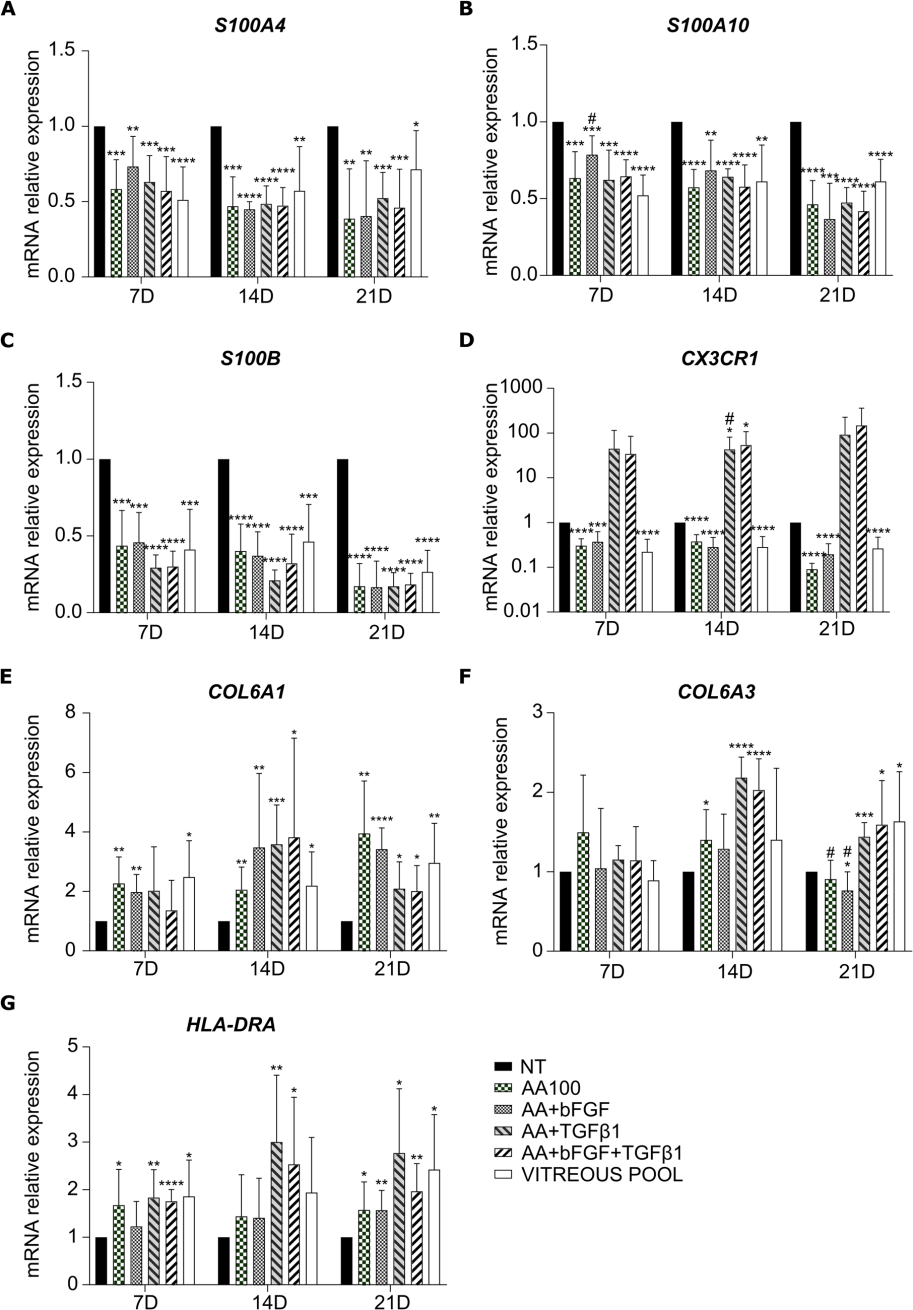
Characterization of hyalocyte gene expression. qRT-PCR analysis of *S100A4* (**a**), *S100A10* (**b**), *S100B* (**c**), *CX3CR1* (**d**), *COL6A1* (**e**), *COL6A3* (**f**), and *HLA-DRA* (**g**) in macrophages treated with ascorbic acid alone or in combination with bFGF and/or TGF-β1, or with a pool of vitreous bodies. Data at day 7, 14, and 21 were normalized to the corresponding NT cells. The bars represent the mean ± standard deviation (*n* = 4: BJ *n* = 3 and Episomal *n* = 1). * and #*p* < 0.05, **p < 0.01, ***p < 0.001, ****p < 0.0001; * vs NT, # vs vitreous pool

We found that *S100A4*, *S100A10*, and *S100B* were significantly downregulated at all the timepoints, as compared to NT, following all treatments, similarly to cells cultured in the presence of the vitreous pool (Fig. 5a, b, and c), whereas *COL6A1* was upregulated, especially at day 14 and 21 (Fig. 5e). *COL6A3* showed a more variable trend, with no significant difference at day 7, upregulation only with AA, AA + TGF-β1, and AA + bFGF + TGF-β1 at day 14, and upregulation with AA + TGF-β1, AA + bFGF + TGF-β1, and the vitreous pool at day 21, while it was slightly downregulated with AA + bFGF (Fig. 5f).

**Fig. 6:**
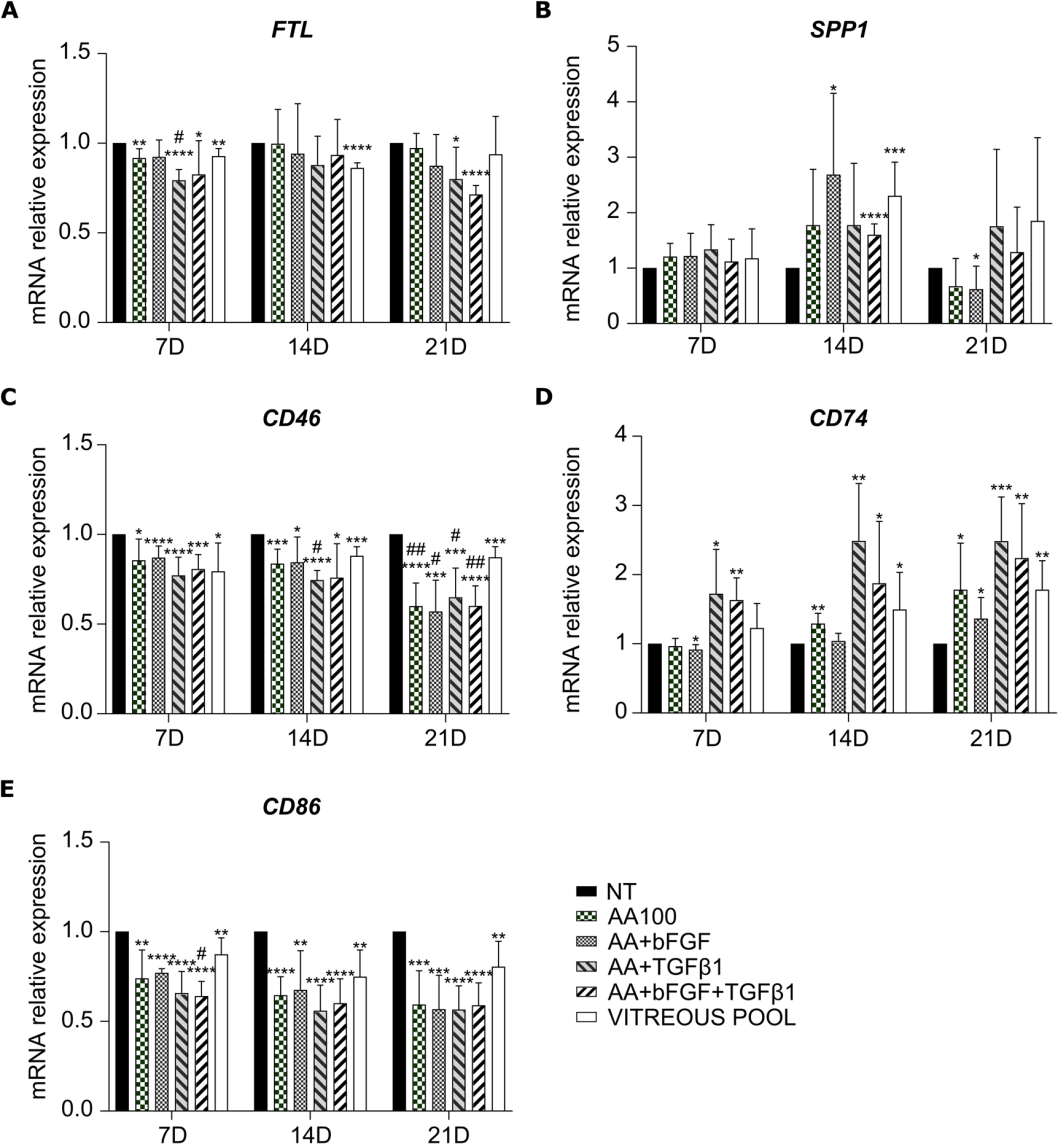
Characterization of hyalocyte gene expression. qRT-PCR analysis of *FTL* (**a**), *SPP1* (**b**), *CD46* (**c**), *CD74* (**d**), and *CD86* (**e**) in macrophages treated with ascorbic acid alone or in combination with bFGF and/or TGF-β1, or with a pool of vitreous bodies. Data at day 7, 14, and 21 were normalized to the corresponding NT cells. The bars represent the mean ± standard deviation (*n* = 4: BJ *n* = 3 and Episomal *n* = 1). * and #*p* < 0.05, ** and ##*p* < 0.01, ***p < 0.001, ****p < 0.0001; * vs NT, # vs vitreous pool

*CX3CR1*, the marker used by Boneva for FACS-sorting [32], was significantly downregulated following AA, AA + bFGF, and vitreous pool treatments; on the contrary, it showed a trend of upregulation in the presence of TGF-β1 (Fig. 5d). Among *HLA-DRA*, *FTL*, *SPP1*, *CD46*, *CD74*, and *CD86*, only *HLA-DRA* and *CD74* were found upregulated, more remarkably at day 21 (Figs. 5g and 6d), while *CD46* and *CD86* resulted slightly, yet significantly, downregulated at all the timepoints, following all the treatments (Fig. 6c and e). Finally, *FTL* was slightly downregulated only at day 7 (Fig. 6a).

### iPSC-derived hyalocytes showed a different protein expression of S100B, CD14, CD49d, and HLA-DR in comparison to NT macrophages

Since S100B resulted the most downregulated gene among the tested ones, its characterization was deepened also at protein level.

**Fig. 7:**
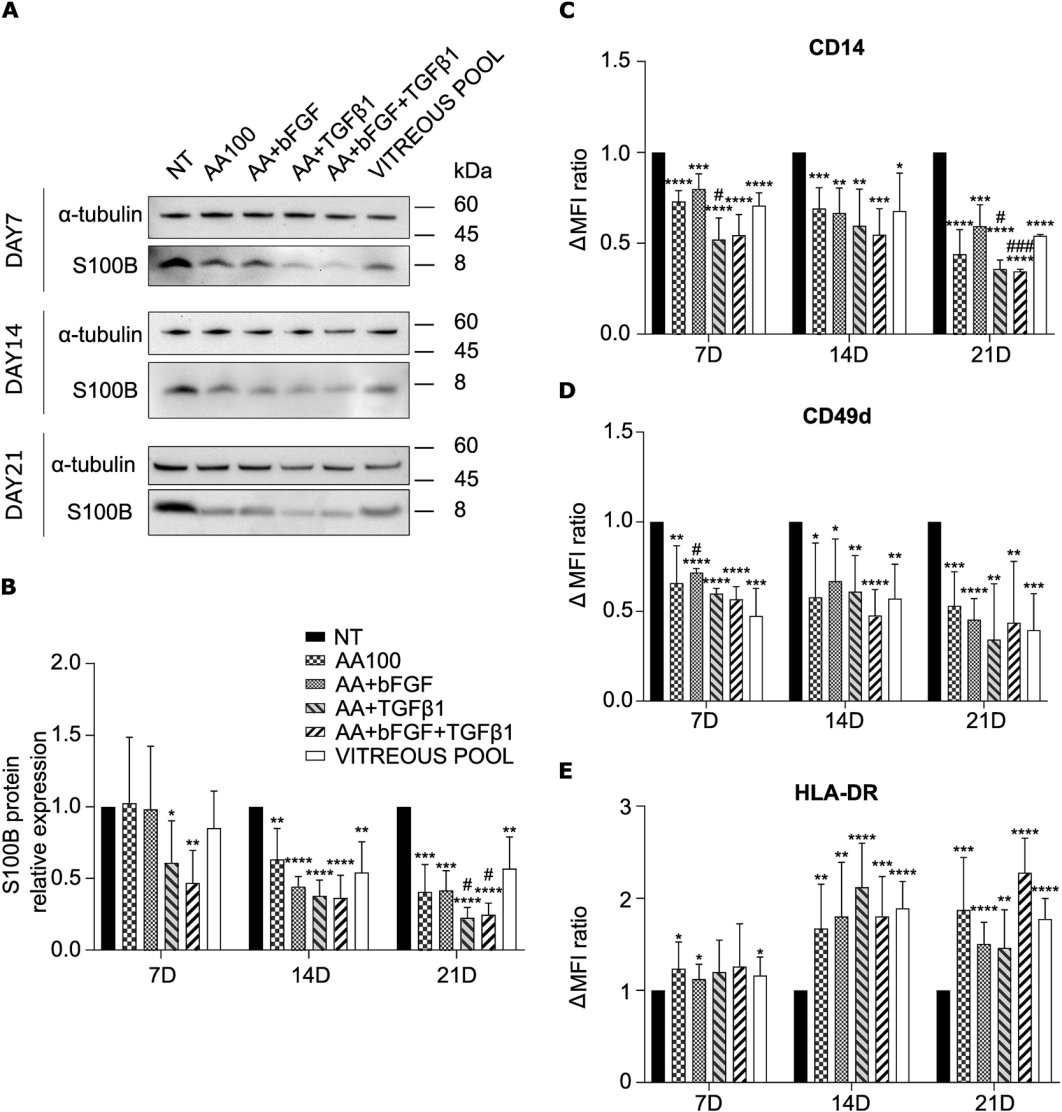
Characterization of hyalocyte protein expression. **a, b** Western blot analysis of S100B expression in macrophages treated with ascorbic acid alone or in combination with bFGF and/or TGF-β1, or with a pool of vitreous bodies, for 7, 14, or 21 days. In panel **a**, representative blots are shown. S100B signal was normalized to α-tubulin. In panel **b**, the bars represent the mean ratio vs NT cells ± standard deviation (*n* = 4: BJ *n* = 3 and Episomal *n* = 1). * and # *p* < 0.05, **p < 0.01, ***p < 0.001, ****p < 0.0001; * vs NT, # vs vitreous pool. **c, d, e** Flow cytometry analysis of CD14 (**c**), CD49d (**d**), and HLA-DR (**e**) expression in macrophages treated with ascorbic acid alone or in combination with bFGF and/or TGF-β1, or with a pool of vitreous bodies, for 7, 14, or 21 days. Data were expressed as the difference between the mean fluorescence intensity (Δ MFI) of stained and unstained cells and were normalized to the corresponding NT cells. The bars represent the mean ± standard deviation (*n* = 3: BJ *n* = 2 and Episomal *n* = 1). * and # *p* < 0.05, **p < 0.01, *** and ### *p* < 0.001, ****p < 0.0001; * vs NT, # vs vitreous pool

S100B protein presented a ubiquitous localization in immunofluorescence staining. The fluorescent signal was strong in all the analyzed samples, irrespectively of the treatment (Suppl. Figure 4). However, immunofluorescence does not provide any quantitative information. On the contrary, Western Blot analysis confirmed S100B downregulation also at protein level, following all treatments, as compared to NT, at day 14 and day 21 (Fig. 7a and b).

Flow cytometry analysis was performed on some typical macrophage surface markers (CD45, CD14, CD11b, CD49d, and HLA-DR). We found that all the cells were CD45-positive (data not shown). Moreover, a significant downregulation of CD14 and CD49d was observed following all treatments, as compared to NT, especially at day 21 (Fig. 7 c and d), while HLA-DR was significantly upregulated (Fig. 7 e).

**Fig. 8:**
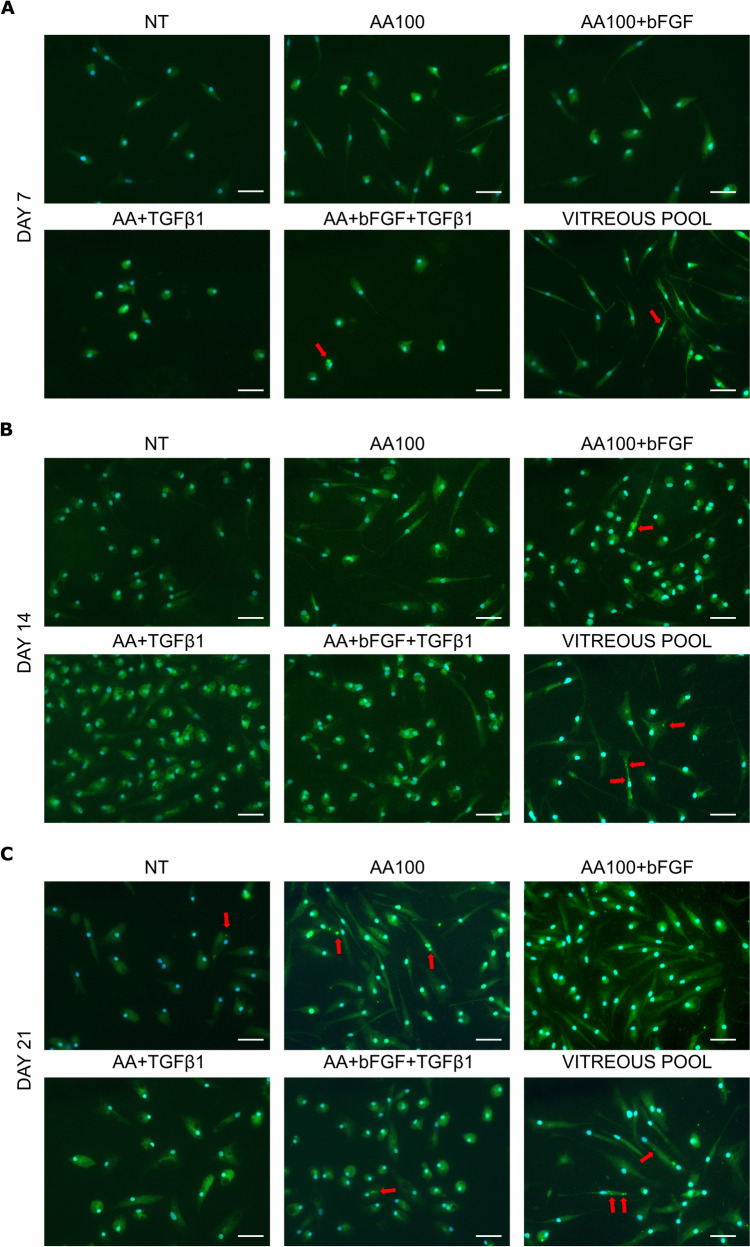
Collagen VI immunofluorescence staining. Macrophages either not treated (NT) or treated with ascorbic acid (AA100) alone or in combination with bFGF and/or TGF-β1, or with a pool of vitreous bodies were stained at day 7 (**a**), 14 (**b**), and 21 (**c**). In green: Collagen VI; in blue, nuclei were stained with DAPI. Red arrows indicate spots with protein accumulation. Scale bar: 50 µm

To demonstrate functional characteristics of the cells, such as the production of collagen, an immunofluorescence was conducted to detect the spatial expression of collagen VI. A diffuse, cytoplasmic staining was detectable in all the samples, including NT, indicating that all the cells could produce and, eventually, secrete procollagen. However, the signal was more intense following all treatments. In particular, protein accumulations in some cytoplasmic spots were observed in some cells, arguably corresponding to secretory vesicles (Fig. 8).

## Discussion

Hyalocytes play both physiological and pathological roles at the vitreoretinal interface, however, there is limited knowledge about the characteristics and functions of these cells. Indeed, to study hyalocytes of human origin, invasive surgery is needed; moreover, pools of eyes are often necessary due to the small number of cells that could be isolated.

Therefore, iPSCs could be a useful tool to obtain an unlimited number of hyalocytes that retain the genetic features of each individual, to allow a better understanding of the cellular origins of some disorders, such as floaters [45, 46], macular pucker, [19, 20] macular holes [21], and proliferative diabetic retinopathy [22].

To our knowledge, no one has ever differentiated hyalocytes from iPSCs, even though macrophage differentiation is well established; moreover, only few reports describe methods to culture hyalocytes [14, 24, 29]. Specifically, Sommer demonstrated that the addition of AA in the culture medium (range 0.1–200 μg/mL) promoted proliferation and collagen production in porcine hyalocytes [29]. Basic FGF (range 0.1–100 ng/mL) increased proliferation and reduced ECM deposition, while TGF-β1 (range 0.1–20 ng/mL) promoted ECM deposition, but decreased cell proliferation rate [14]. More recently, Nuzzi managed to isolate and culture human hyalocytes from vitrectomies in the presence of 10 ng/mL bFGF, 5 ng/mL TGF-β1, 10 ng/mL PDGF-BB, 200 µg/mL AA, 1 mg/mL dexamethasone, or 5 µM H_2_O_2_: only bFGF could enhance cell proliferation and hyaluronan production [31].

In the present work, we set up a 54-day protocol to differentiate human iPSCs into hyalocytes. First, a commercial kit was used to obtain HSPCs without requiring extensive manipulation. Cells collected from culture supernatant expressed CD45, CD34, and CD43 and appeared as blast cells at a morphological evaluation, showing that they were *bona fide* HSPCs [35].

Then, HSPCs were stimulated with M-CSF to allow monocyte-macrophage differentiation. From literature, a 10-to-14-day differentiation was recommended [34]. To this reason, we characterized cells after 14 days of culture, but the differentiation was also extended for an additional week. Cells showed a morphology similar to PBMC-derived macrophages, a finding that was corroborated through cytological inspection, and presented the typical macrophage surface markers [47]. Moreover, the upregulation of a group of macrophage-expressed genes [48] was observed after 14 days, while an even more pronounced upregulation was recorded at day 21. Therefore, it can be reasonably excluded that the obtained cells are mesenchymal stem cells or fibroblasts.

Based on the previous literature, we treated 21-day macrophages with AA alone or in combination with bFGF and/or TGF-β1 for hyalocyte differentiation. AA has already been used for mesenchymal stem cell differentiation into chondrocytes, osteoblasts, and tendons, beside for hyalocyte culture [49]. As positive control of differentiation, we cultured cells in the presence of a vitreous pool from vitrectomies to simulate the environment in which real hyalocytes reside. The vitreous pool was diluted 1:4 in culture medium, since it represented the highest dilution to retain vitreous fluid biological effects [44]. In this way, the advantage was that all the cells shared the same genetic background. We employed qRT-PCR to analyze collagen and HAS mRNA expression in macrophages treated with the vitreous pool, enabling a comparison of their profiles with those of in situ vitreous cells from three patients: similar patterns were observed, with any differences potentially arising from the extensive in vitro culture of macrophages, which is lacking in in situ cells. This suggests that we can trust this model for hyalocyte differentiation.

At microscopy inspection, we observed a similar morphology among vitreous-treated cells and AA- or AA + bFGF-treated cells: specifically, cells were more elongated than NT, while TGF-β1-treated cells were smaller and round-shaped, probably due to detrimental effects of extended treatments with this cytokine that can also induce senescence and apoptosis [50]. Previous works demonstrated that the tested treatments had different impacts on actin filaments organization and thickness [14, 31]. However, in our setting, phalloidin staining could not highlight any remarkable difference, but the staining was still useful to confirm the different morphologies.

Since a consensus on hyalocytes markers is lacking, the expression of typical macrophage surface markers was checked after treatments. Indeed, a previous work stated that the most classical macrophage marker, CD14, was expressed also by hyalocytes, together with CD11b, CD68, CD204, and CD206 [9]; on the contrary, hyalocytes negative for CD14, CD11b, and CD68 and positive for CD45, CD11a, and CD64 were depicted elsewhere [30]. We found that all the cells were CD45-positive, and that all the markers previously used to confirm macrophage differentiation were expressed also by treated cell; however, CD14 and CD49d were significantly downregulated following all treatments and in vitreous-treated macrophages, as compared to NT, while HLA-DR was upregulated, especially at day 14 and day 21.

To further characterize treated macrophages, several genes that have been variably claimed to be up or downregulated in hyalocytes in comparison to macrophages were analyzed. Some categories have been taken into account: S100 family, ECM production, and the genes that Boneva found upregulated in hyalocytes [32].

S100-negative hyalocytes were described in horse [5], quail [8], pig [12], and human [9]; on the contrary, S100 proteins have been found positive in bovine hyalocytes [15, 23] and still in human [30]. Among the S100 family members, *S100A4*, *S100A10*, and above all *S100B* were downregulated following all treatments, especially at longer timepoints; S100B downregulation was also confirmed at the protein level. Nonetheless, it must be stated that mRNAs and the protein were still present in all the treated macrophages that could not be defined as S100-negative.

For ECM production, the genes for the main vitreous collagens (*COL2A1*, *COL6A1*, *COL6A3*, *COL9A1*, and *COL11A1*) and for three hyaluronan synthase isoforms (*HAS1*, *HAS2*, and *HAS3*) were analyzed. Kashiwagi [9] described the expression of *COL2A1*, *HAS1*, and *HAS3* in human transformed hyalocytes; in our hands, *COL9A1* and *COL11A1* were not detectable, while *COL2A1*, *HAS1*, *HAS2*, and *HAS3* were markedly low expressed. Interestingly, among the genes encoding for collagen VI, a minor component of vitreous body, but responsible for the spacing among collagen fibers together with collagen IX [51, 52], *COL6A1* resulted upregulated at longer timepoints. Its expression was also confirmed at the protein level by immunofluorescence. Collagen VI secretion has been previously described in nondestructive, matrix-conserving macrophages; additionally, it has been demonstrated that its synthesis in macrophages is strongly enhanced by TGF-β1 [53]. In our experimental setting, all the cells stained positive for collagen VI; however, a more intense signal was collected in the cytoplasm of treated cells, thereby confirming the gene expression data. Some cells exhibited the presence of concentrated areas of staining, presumably corresponding to Golgi cisternae or secretory vesicles.

Boneva [32] FACS-sorted hyalocytes as CD45^+^CD11b^+^CX3CR1^+^Mat-Mac^+^ cells and analyzed them by RNA-seq, finding that *SPP1*, *FTL*, *CD74*, *HLA-DRA*, *POMC*, *CD46*, and *CD86*, were upregulated in comparison to monocytes, macrophages, and microglia.

Interestingly, in our setting, only TGF-β1 induced *CX3CR1* upregulation, as well known from literature [54, 55], while it was significantly downregulated following all the other treatments. *POMC* was not detectable, while *HLA-DRA* and *CD74* were found upregulated. Finally, *CD46, FTL,* and *CD86* resulted slightly downregulated following all the treatments; however, all the three genes were so highly expressed that the biological meaning of their downregulation is not clear.

In conclusion, to the best of the Authors’ knowledge, this is the first report of human iPSC differentiation into *bona fide* hyalocytes. We took advantage of an intermediate macrophage differentiation, followed by 3 weeks of different treatments. After 21 days, AA-treated macrophages presented a morphology, gene, and protein expression similar to macrophages cultured with vitreous pool, while bFGF did not exert any significant additive effect. On the contrary, TGF-β1 negatively impacted on both morphology and *CX3CR1* expression.

Altogether, hyalocytes could be obtained by treating iPSC-derived macrophages with AA for 21 days. However, in the present work, cell characterization was limited to genes and markers already explored in the existing, skimpy literature; since the emergence of unexpected targets cannot be excluded, in the future, more comprehensive approaches, such as transcriptomics, will be necessary to deepen iPSC-derived hyalocyte characterization.

## Supplementary Information

Below is the link to the electronic supplementary material.

Supplementary file1 (PDF 1191 KB)

## Data Availability

Not applicable.

## References

[1] Bishop PN. Structural macromolecules and supramolecular organisation of the vitreous gel. Prog Retin Eye Res. 2000;19:323–44. 10749380 10.1016/s1350-9462(99)00016-6

[2] Donati S, Caprani SM, Airaghi G, Vinciguerra R, Bartalena L, Testa F, et al. Vitreous substitutes: the present and the future. Biomed Res Int. 2014;2014: 351804. 24877085 10.1155/2014/351804 PMC4024399

[3] Ankamah E, Sebag J, Ng E, Nolan JM. Vitreous antioxidants, degeneration, and vitreo-retinopathy: exploring the links. Antioxidants. 2020;9(1):7. 10.3390/antiox9010007 PMC7022282 31861871

[4] Grabner G, Boltz G, Forster O, Grabner G. Macrophage-like properties of human hyalocytes. Invest Ophthalmol Vis Sci. 1980;19:333–40. 7358486

[5] Sano Y, Matsuda K, Okamoto M, Takehana K, Hirayama K, Taniyama H. Characterization of equine hyalocytes: their immunohistochemical properties, morphologies and distribution. J Vet Med Sci. 2016;78:937–42. 26888584 10.1292/jvms.15-0511 PMC4937152

[6] Qiao H, Hisatomi T, Sonoda KH, Kura S, Sassa Y, Kinoshita S, et al. The characterisation of hyalocytes: the origin, phenotype, and turnover. Br J Ophthalmol. 2005;89:513–7. 15774935 10.1136/bjo.2004.050658 PMC1772586

[7] Rosmus D-D, Koch J, Hausmann A, Chiot A, Arnhold F, Masuda T, et al. Redefining the ontogeny of hyalocytes as yolk sac-derived tissue-resident macrophages of the vitreous body. J Neuroinflammation. 2024;21:168. 38961498 10.1186/s12974-024-03110-x PMC11223341

[8] Llombart C, Nacher V, Ramos D, Luppo M, Carretero A, Navarro M, et al. Morphological characterization of pecteneal hyalocytes in the developing quail retina. J Anat. 2009;215:280–91. 19566699 10.1111/j.1469-7580.2009.01117.x PMC2750761

[9] Kashiwagi Y, Nishitsuka K, Takamura H, Yamamoto T, Yamashita H. Cloning and characterization of human vitreous tissue-derived cells. Acta Ophthalmol. 2011;89:538–43. 19878119 10.1111/j.1755-3768.2009.01736.x

[10] Vagaja NN, Chinnery HR, Binz N, Kezic JM, Rakoczy EP, McMenamin PG. Changes in murine hyalocytes are valuable early indicators of ocular disease. Investig Ophthalmol Vis Sci. 2012;53:1445–51. 22297487 10.1167/iovs.11-8601

[11] Newsome DA, Linsenmayer TF, Trelstad RL. Vitreous body collagen: evidence for a dual origin from the neural retina and hyalocytes. J Cell Biol. 1976;71:59–67. 977655 10.1083/jcb.71.1.59 PMC2109724

[12] Nishitsuka K, Kashiwagi Y, Tojo N, Kanno C, Takahashi Y, Yamamoto T, et al. Hyaluronan production regulation from porcine hyalocyte cell line by cytokines. Exp Eye Res. 2007;85:539–45. 17707370 10.1016/j.exer.2007.07.006

[13] Osterlin SE, Jacobson B. The synthesis of hyaluronic acid in vitreous. I. Soluble and particulate transferases in hyalocytes. Exp Eye Res. 1968;7:497–510. 5716094 10.1016/s0014-4835(68)80003-x

[14] Sommer F, Pollinger K, Brandl F, Weiser B, Teßmar J, Blunk T, et al. Hyalocyte proliferation and ECM accumulation modulated by bFGF and TGF-β1. Graefe’s Arch Clin Exp Ophthalmol. 2008;246:1275–84. 18461346 10.1007/s00417-008-0846-z

[15] Noda Y, Hata Y, Hisatomi T, Nakamura Y, Hirayama K, Miura M, et al. Functional properties of hyalocytes under PDGF-rich conditions. Invest Ophthalmol Vis Sci. 2004;45:2107–14. 15223783 10.1167/iovs.03-1092

[16] Hata Y, Sassa Y, Kita T, Miura M, Kano K, Kawahara S, et al. Vascular endothelial growth factor expression by hyalocytes and its regulation by glucocorticoid. Br J Ophthalmol. 2008;92:1540–4. 18952656 10.1136/bjo.2008.141002

[17] Sakamoto T, Ishibashi T. HYALOCYTES essential cells of the vitreous cavity in vitreoretinal pathophysiology? Retina. 2011;31(2):222–8. 21240043 10.1097/IAE.0b013e3181facfa9

[18] Sonoda K-H, Sakamoto T, Qiao H, Hisatomi T, Oshima T, Tsutsumi-Miyahara C, et al. The analysis of systemic tolerance elicited by antigen inoculation into the vitreous cavity: vitreous cavity-associated immune deviation. Immunology. 2005;116:390–9. 16236129 10.1111/j.1365-2567.2005.02239.x PMC1802422

[19] Schumann RG, Gandorfer A, Ziada J, Scheler R, Schaumberger MM, Wolf A, et al. Hyalocytes in idiopathic epiretinal membranes: a correlative light and electron microscopic study. Graefe’s Arch Clin Exp Ophthalmol. 2014;252:1887–94. 25377434 10.1007/s00417-014-2841-x

[20] Kohno R, Hata Y, Kawahara S, Kita T, Arita R, Mochizuki Y, et al. Possible contribution of hyalocytes to idiopathic epiretinal membrane formation and its contraction. Br J Ophthalmol. 2009;93:1020–6. 19429593 10.1136/bjo.2008.155069

[21] Schumann RG, Eibl KH, Zhao F, Scheerbaum M, Scheler R, Schaumberger MM, et al. Immunocytochemical and ultrastructural evidence of glial cells and hyalocytes in internal limiting membrane specimens of idiopathic macular holes. Investig Ophthalmol Vis Sci. 2011;52:7822–34. 21900375 10.1167/iovs.11-7514

[22] Boneva SK, Wolf J, Hajdú RI, Prinz G, Salié H, Schlecht A, et al. In-depth molecular characterization of neovascular membranes suggests a role for hyalocyte-to-myofibroblast transdifferentiation in proliferative diabetic retinopathy. Front Immunol. 2021;12: 757607. 34795670 10.3389/fimmu.2021.757607 PMC8593213

[23] Hirayama K, Hata Y, Noda Y, Miura M, Yamanaka I, Shimokawa H, et al. The involvement of the rho-kinase pathway and its regulation in cytokine-induced collagen gel contraction by hyalocytes. Invest Ophthalmol Vis Sci. 2004;45:3896–903. 15505034 10.1167/iovs.03-1330

[24] Micera A, Balzamino BO, Cosimi P, Esposito G, Ripandelli G, Rossi T. Short-term culture of human hyalocytes retains their initial phenotype and displays their contraction abilities. Cells. 2024;13:1837. 39594586 10.3390/cells13221837 PMC11592754

[25] Hannover A (1840) Ueber die Netzhaut und ihre Gehirnsubstanz bei Wirbelthieren, mit Ausnahme des Menschen. 1840

[26] Haddad A, André JC. Hyalocyte-like cells are more numerous in the posterior chamber than they are in the vitreous of the rabbit eye. Exp Eye Res. 1998;66:709–18. 9657903 10.1006/exer.1997.0476

[27] Ogawa K. Scanning electron microscopic study of hyalocytes in the guinea pig eye. Arch Histol Cytol. 2002;65:263–8. 12389665 10.1679/aohc.65.263

[28] Sommer F, Brandl F, Weiser B, Teßmar J, Blunk T, Göpferich A. FACS as useful tool to study distinct hyalocyte populations. Exp Eye Res. 2009;88:995–9. 19073178 10.1016/j.exer.2008.11.026

[29] Sommer F, Kobuch K, Brandl F, Wild B, Framme C, Weiser B, et al. Ascorbic acid modulates proliferation and extracellular matrix accumulation of hyalocytes. Tissue Eng. 2007;13:1281–9. 17518733 10.1089/ten.2006.0274

[30] Lazarus HS, Hageman GS. In situ characterization of the human hyalocyte. Arch Ophthalmol (Chicago, Ill 1960). 1994;112:1356–62. 10.1001/archopht.1994.01090220106031 7945040

[31] Nuzzi R, Bergandi L, Zabetta LC, D’Errico L, Riscaldino F, Menegon S, et al. In vitro generation of primary cultures of human hyalocytes. Mol Vis. 2020;26:818–29. 33456301 PMC7803295

[32] Boneva SK, Wolf J, Rosmus D-D, Schlecht A, Prinz G, Laich Y, et al. Transcriptional profiling uncovers human hyalocytes as a unique innate immune cell population. Front Immunol. 2020;11: 567274. 33042148 10.3389/fimmu.2020.567274 PMC7517040

[33] Wolf J, Boneva S, Rosmus D-D, Agostini H, Schlunck G, Wieghofer P, et al. Deciphering the molecular signature of human hyalocytes in relation to other innate immune cell populations. Invest Ophthalmol Vis Sci. 2022;63:9. 35266958 10.1167/iovs.63.3.9 PMC8934546

[34] Merling RK, Sweeney CL, Chu J, Bodansky A, Choi U, Priel DL, et al. An AAVS1-targeted minigene platform for correction of iPSCs from all five types of chronic granulomatous disease. Mol Ther. 2015;23:147–57. 25288370 10.1038/mt.2014.195 PMC4426805

[35] Ruiz JP, Chen G, Haro Mora JJ, Keyvanfar K, Liu C, Zou J, et al. Robust generation of erythroid and multilineage hematopoietic progenitors from human iPSCs using a scalable monolayer culture system. Stem Cell Res. 2019;41: 101600. 31710911 10.1016/j.scr.2019.101600 PMC6953424

[36] Manganelli M, Mazzoldi EL, Ferraro RM, Pinelli M, Parigi M, Aghel SAM, et al. Progesterone receptor is constitutively expressed in induced pluripotent stem cells (iPSCs). Stem cell Rev reports. 2024;20:2303–17. 10.1007/s12015-024-10776-6 PMC11554879 39168923

[37] Ferraro RM, Ginestra PS, Lanzi G, Giliani S, Ceretti E. Production of micro-patterned substrates to direct human iPSCs-derived neural stem cells orientation and interaction. Procedia CIRP. 2017;65:225–30.

[38] Ferraro RM, Ginestra PS, Giliani S, Ceretti E. Carbonization of polymer precursors substrates to direct human iPSC-derived neurons differentiation and maturation. Procedia CIRP. 2020;89:39–44.

[39] Itoh M, Umegaki-Arao N, Guo Z, Liu L, Higgins CA, Christiano AM. Generation of 3D skin equivalents fully reconstituted from human induced pluripotent stem cells (iPSCs). PLoS One. 2013;8:e77673. 24147053 10.1371/journal.pone.0077673 PMC3795682

[40] Osaki T, Uzel SGM, Kamm RD. Microphysiological 3D model of amyotrophic lateral sclerosis (ALS) from human iPS-derived muscle cells and optogenetic motor neurons. Sci Adv. 2018;4:1–16. 10.1126/sciadv.aat5847 PMC6179377 30324134

[41] Canosa S, Bergandi L, Macrì C, Charrier L, Paschero C, Carosso A, et al. Morphokinetic analysis of cleavage stage embryos and assessment of specific gene expression in cumulus cells independently predict human embryo development to expanded blastocyst: a preliminary study. J Assist Reprod Genet. 2020;37:1409–20. 32436046 10.1007/s10815-020-01806-6 PMC7311629

[42] Revelli A, Canosa S, Bergandi L, Skorokhod OA, Biasoni V, Carosso A, et al. Oocyte polarized light microscopy, assay of specific follicular fluid metabolites, and gene expression in cumulus cells as different approaches to predict fertilization efficiency after ICSI. Reprod Biol Endocrinol. 2017;15:47. 28645283 10.1186/s12958-017-0265-2 PMC5481970

[43] Ferraro RM, Masneri S, Lanzi G, Barisani C, Piovani G, Savio G, et al. Establishment of three iPSC lines from fibroblasts of a patient with Aicardi Goutieres syndrome mutated in RNaseH2B. Stem Cell Res. 2019;41: 101620. 31678772 10.1016/j.scr.2019.101620

[44] Rezzola S, Guerra J, Krishna Chandran AM, Loda A, Cancarini A, Sacristani P, et al. VEGF-independent activation of müller cells by the vitreous from proliferative diabetic retinopathy patients. Int J Mol Sci. 2021;22(4):2179. 33671690 10.3390/ijms22042179 PMC7926720

[45] Riva L, Mazzoldi EL, Ginestra PS, Ceretti E, Giliani SC. Eye model for floaters’ studies: production of 3D printed scaffolds. Prog Addit Manuf. 2022;7(6):1127-1140.

[46] Mazzoldi EL, Riva L, Ferraro RM, Ginestra PS, Giliani SC. 3D printing of biocompatible scaffolds for eye tissue engineering. Procedia CIRP. 2022;110:214–9.

[47] Kelly A, Grabiec AM, Travis MA. Culture of human monocyte-derived macrophages. Methods Mol Biol. 2018;1784:1–11. 29761383 10.1007/978-1-4939-7837-3_1

[48] Mukherjee C, Hale C, Mukhopadhyay S. A simple multistep protocol for differentiating human induced pluripotent stem cells into functional macrophages. Methods Mol Biol. 2018;1784:13–28. 29761384 10.1007/978-1-4939-7837-3_2

[49] D’Aniello C, Cermola F, Patriarca EJ, Minchiotti G. Vitamin C in stem cell biology: impact on extracellular matrix homeostasis and epigenetics. Stem Cells Int. 2017;1:8936156. 10.1155/2017/8936156 PMC5415867 28512473

[50] Zhang Y, Alexander PB, Wang X-F. TGF-β family signaling in the control of cell proliferation and survival. Cold Spring Harb Perspect Biol. 2017;9(4):a022145. 27920038 10.1101/cshperspect.a022145 PMC5378054

[51] Bishop P, Ayad S, Reardon A, McLeod D, Sheehan J, Kielty C. Type VI collagen is present in human and bovine vitreous. Graefe’s Arch Clin Exp Ophthalmol. 1996;234:710–3. 8950592 10.1007/BF00292358

[52] Bishop PN, Holmes DF, Kadler KE, McLeod D, Bos KJ. Age-related changes on the surface of vitreous collagen fibrils. Invest Ophthalmol Vis Sci. 2004;45:1041–6. 15037566 10.1167/iovs.03-1017

[53] Schnoor M, Cullen P, Lorkowski J, Stolle K, Robenek H, Troyer D, et al. Production of type VI collagen by human macrophages: a new dimension in macrophage functional heterogeneity. J Immunol. 2008;180:5707–19. 18390756 10.4049/jimmunol.180.8.5707

[54] Chen S, Luo D, Streit WJ, Harrison JK. TGF-beta1 upregulates CX3CR1 expression and inhibits fractalkine-stimulated signaling in rat microglia. J Neuroimmunol. 2002;133:46–55. 12446007 10.1016/s0165-5728(02)00354-5

[55] Helmke A, Nordlohne J, Balzer MS, Dong L, Rong S, Hiss M, et al. CX3CL1-CX3CR1 interaction mediates macrophage-mesothelial cross talk and promotes peritoneal fibrosis. Kidney Int. 2019;95:1405–17. 30948201 10.1016/j.kint.2018.12.030

