# Supplementary material for: Differentiation of human hyalocytes from induced pluripotent stem cells through ascorbic acid treatment
Source: Hum Cell. 2025 Feb 12;38(2):52. doi: 10.1007/s13577-025-01182-2 (PMC11821750; doi:10.1007/s13577-025-01182-2)

Differentiation of human hyalocytes from induced pluripotent stem cells through ascorbic acid treatment

Elena Laura Mazzoldi [a,b,*], Gabriele Benini [a,b], Rosalba Monica Ferraro [a,b], Moira Micheletti [c], Giovanni Martellosio [c], Viola Balduchelli [b], Piergiuseppe Sacristani [d], Daniele Lussignoli [d], Francesco Semeraro [d], Sara Rezzola [a], Marco Presta [a], Loredana Bergandi [e], Alessandro Meduri [f], Silvia Clara Giliani [a,b,g,h]

a) Department of Molecular and Translational Medicine, University of Brescia, Viale Europa 11, 25123 Brescia, Italy

b) «Angelo Nocivelli» Institute for Molecular Medicine, ASST Spedali Civili, Piazzale Spedali Civili 1, 25123 Brescia, Italy

c) Hematology Unit, Clinical Chemistry Laboratory, Diagnostic Department, ASST Spedali Civili of Brescia, Piazzale Spedali Civili 1, 25123 Brescia, Italy

d) Department of Ophthalmology, University of Brescia, ASST Spedali Civili, Piazzale Spedali Civili 1, 25123 Brescia, Italy

e) Department of Oncology, University of Torino, Via Santena 5 bis, 10126 Torino, Italy

f) Ophthalmology Clinic, Department of Biomedical and Dental Sciences and Morphofunctional Imaging, University of Messina, 98125 Messina, Italy

g) Section of Medical Genetics and Cytogenetics, ASST Spedali Civili of Brescia, Piazzale Spedali Civili 1, 25123 Brescia, Italy;

h) National Center for Gene Therapy and Drugs based on RNA Technology – CN3, Brescia, Italy

* Correspondence to Elena Laura Mazzoldi (ORCID 0000-0001-8488-7750): elena.mazzoldi@unibs.it

SUPPLEMENTARY INFORMATION

SUPPLEMENTARY MATERIALS AND METHODS

Immunofluorescence

For phalloidin staining, cells seeded on coverslips were fixed and permeabilized with FIX & PERM® kit (Nordic MUbio, Susteren, The Netherlands), and blocked with 5% BSA in PBS; then, Alexa Fluor™ 488 phalloidin (1:400, Molecular Probes, Thermo Fisher Scientific) and 100 ng/mL DAPI (Sigma Aldrich) have been added for 15 min in the dark.

For S100B staining, fixed cells underwent heat-induced antigen retrieval in hot 10 mM citrate buffer, pH 6, for 20 min. Then, cells were permeabilized in 0.2 % Triton X-100 in PBS and blocked in 5% BSA in PBS. Cells have been incubated with primary antibody, mouse anti-human S100B 1:50 (Santa Cruz Biotechnology, Dallas, TX) in 5% BSA in PBS for 2h at room temperature, then with secondary antibody Alexa Fluor™ 488 goat anti-mouse IgG (H+L) 1:300 (Invitrogen, Thermo Fisher Scientific) and 100 ng/mL DAPI for 1h in the dark.

Coverslips were mounted with ProLong™ Gold Antifade mountant (Invitrogen, Thermo Fisher Scientific), and were observed with an Olympus IX70 inverted fluorescence microscope (Olympus, Tokyo, Japan). Images were acquired by using Image-Pro Plus v7.0 software (Media Cybernetics, Rockville, MD).

SUPPLEMENTARY FIGURES

Supplementary Figure 1. Phalloidin fluorescence staining of macrophages either not treated (NT) or treated with ascorbic acid (AA100) alone or in combination with bFGF and/or TGFβ1, or with a pool of vitreous bodies. Figure 1 reports cells at day 7 of treatment. In green, F-actin was stained with phalloidin, in blue, nuclei were stained with DAPI. **A)** 60x magnification; **B)** 100x magnification. Scale bar: 20 μm.

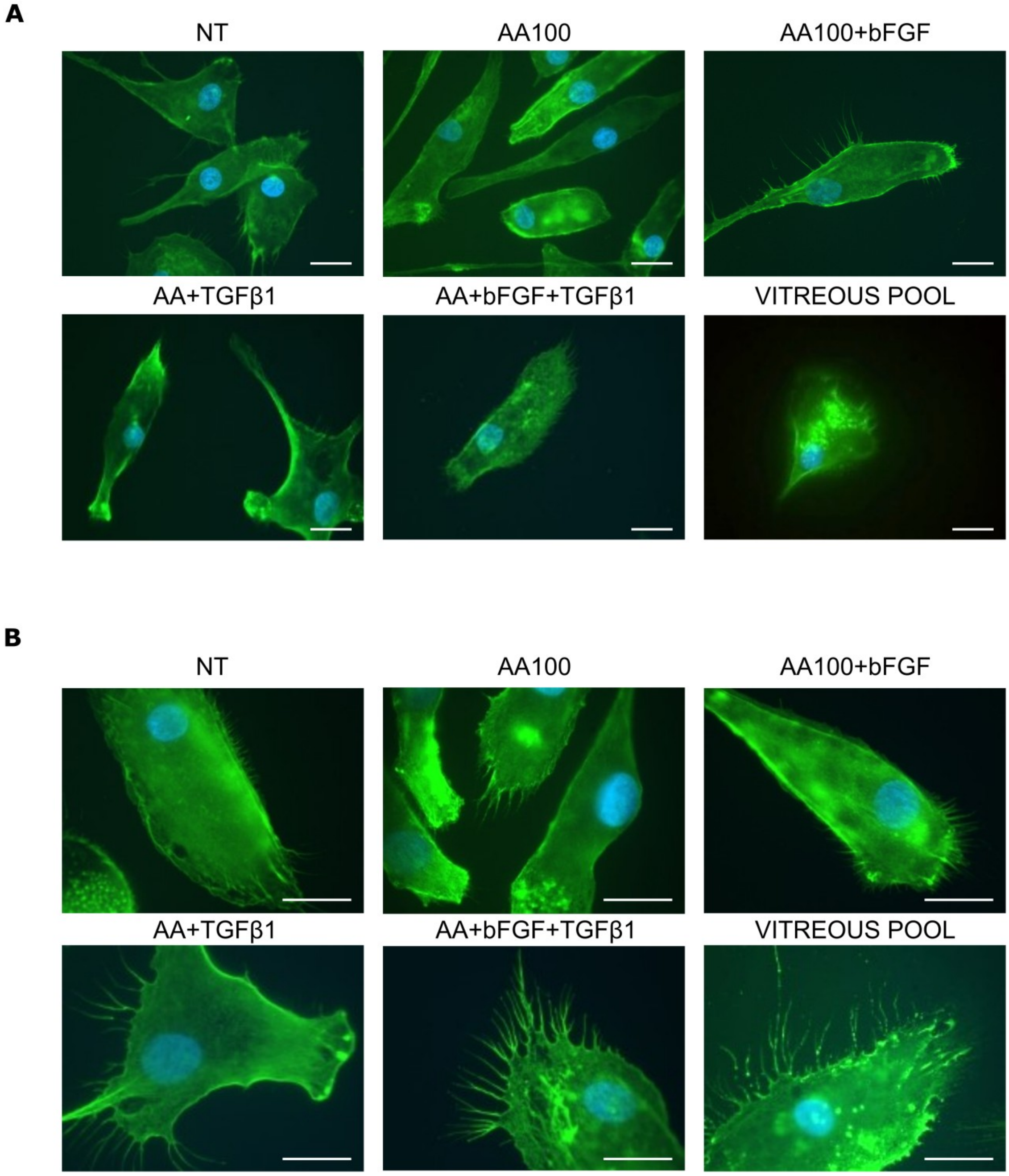

Supplementary Figure 2. Phalloidin fluorescence staining of macrophages either not treated (NT) or treated with ascorbic acid (AA100) alone or in combination with bFGF and/or TGFβ1, or with a pool of vitreous bodies. Figure 2 reports cells at day 14 of treatment. In green, F-actin was stained with phalloidin, in blue, nuclei were stained with DAPI. **A)** 60x magnification; **B)** 100x magnification. Scale bar: 20 µm.

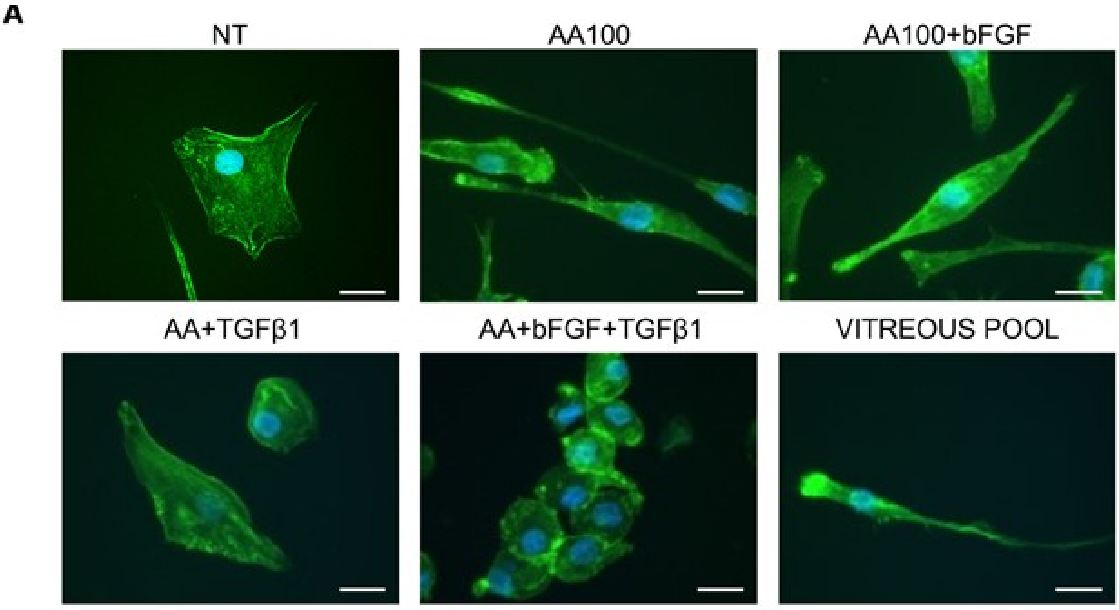

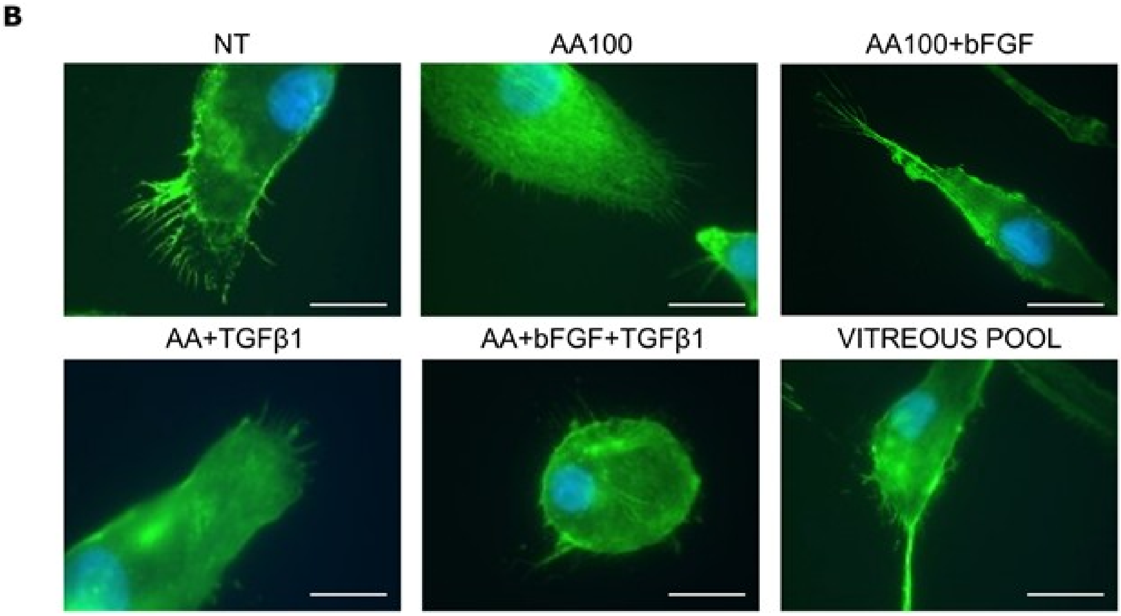

Supplementary Figure 3. Phalloidin fluorescence staining of macrophages either not treated (NT) or treated with ascorbic acid (AA100) alone or in combination with bFGF and/or TGFβ1, or with a pool of vitreous bodies. Figure 3 reports cells at day 21 of treatment. In green, F-actin was stained with phalloidin, in blue, nuclei were stained with DAPI. **A)** 60x magnification; **B)** 100x magnification. Scale bar: 20 µm.

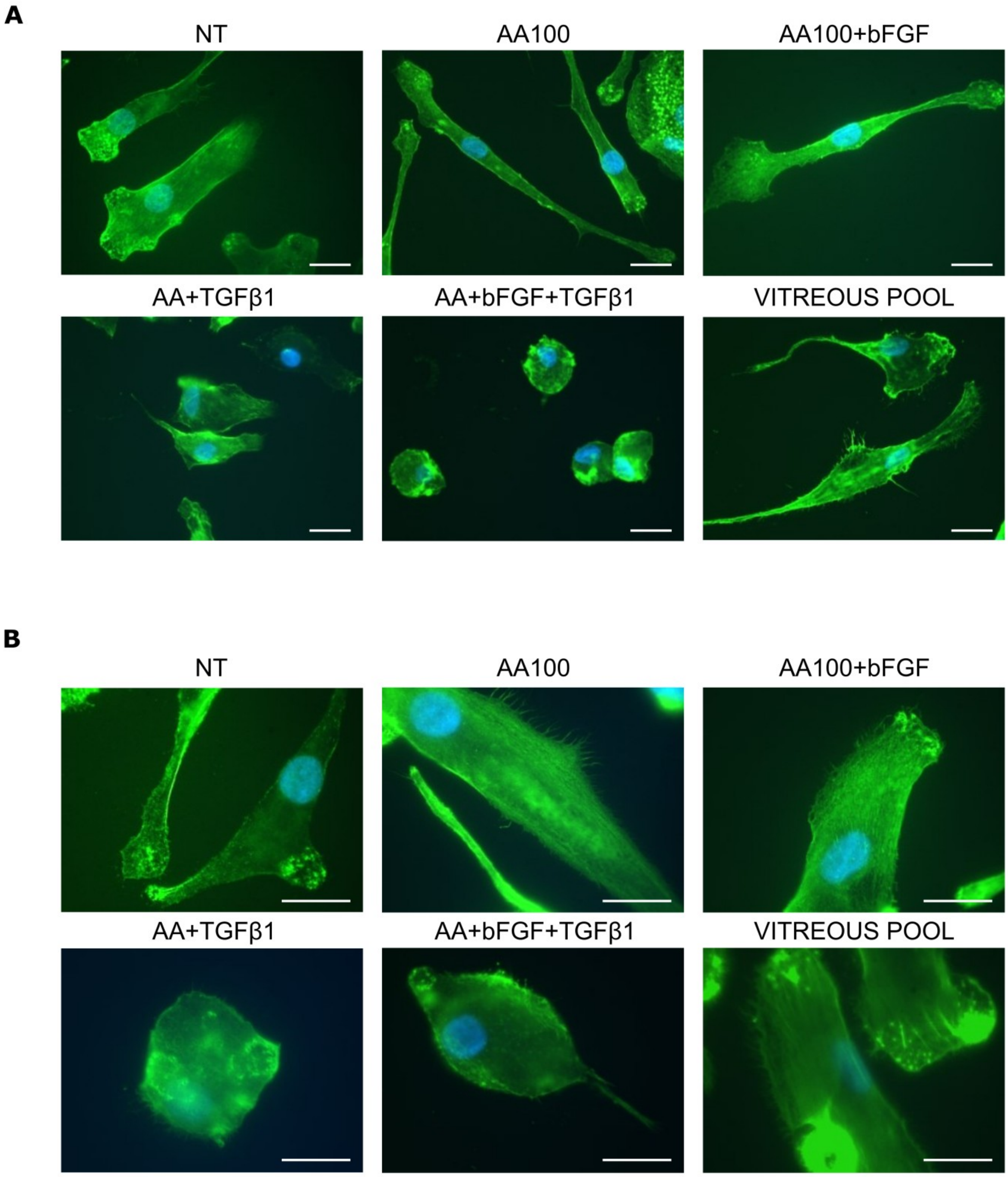

Supplementary Figure 4. S100B immunofluorescence staining. Macrophages either not treated (NT) or treated with ascorbic acid (AA100) alone or in combination with bFGF and/or TGFβ1, or with a pool of vitreous bodies were stained at day 7 (**A**), 14 (**B**), and 21 (**C**). In green: S100B; in blue, nuclei were stained with DAPI. Scale bar: 50 μm.

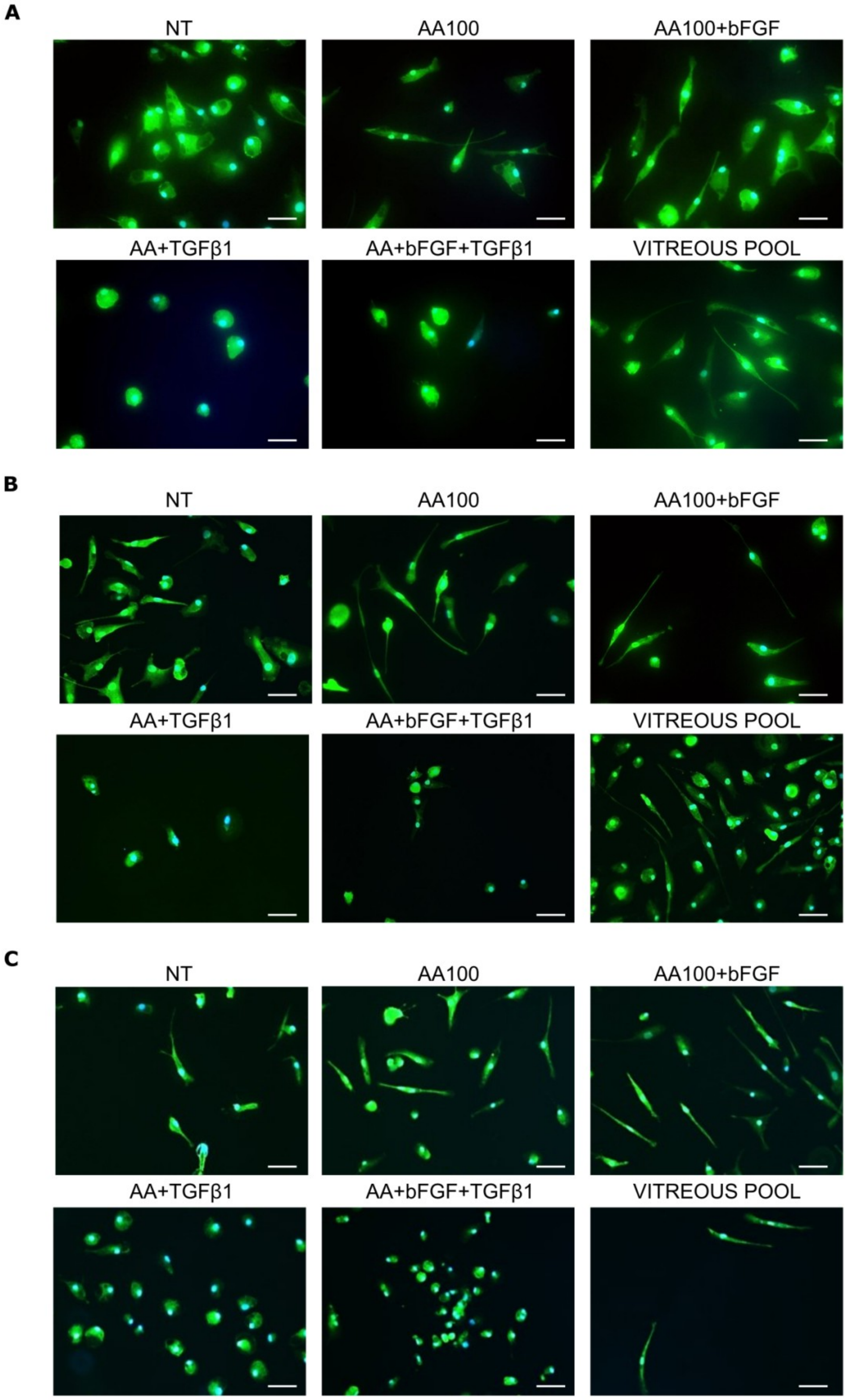

Supplement: Supplementary file 1 — Supplementary file1 (PDF 1191 KB) [file 13577_2025_1182_MOESM1_ESM.pdf]
